# A Single‐Nucleus Transcriptomic Atlas Reveals Cellular and Genetic Characteristics of Alzheimer's‐Like Pathology in Aging Tree Shrews

**DOI:** 10.1002/mco2.70114

**Published:** 2025-03-15

**Authors:** Liu‐Lin Xiong, Rong He, Ruo‐Lan Du, Rui‐Ze Niu, Lu‐Lu Xue, Li Chen, Li‐Ren Huangfu, Qiu‐Xia Xiao, Jing Li, Yong‐Ping Li, Si‐Min Zhang, Chang‐Yin Yu, Xiao‐He Tian, Ting‐Hua Wang

**Affiliations:** ^1^ Department of Anesthesiology The First People's Hospital of Zunyi (The Third Affiliated Hospital of Zunyi Medical University) Zunyi Guizhou China; ^2^ Institute of Neuroscience Kunming Medical University Kunming Yunnan China; ^3^ Mental Health Center of Kunming Medical University Kunming Yunnan China; ^4^ Institute of Neurological Disease, National‐Local Joint Engineering Research Center of Translational Medicine, State Key Lab of Biotherapy West China Hospital Sichuan University Chengdu Sichuan China; ^5^ Yunnan Key Laboratory of Primate Biomedical Research, Institute of Primate Translational Medicine Kunming University of Science and Technology Kunming China; ^6^ Department of Neurology Affiliated Hospital of Zunyi Medical University Zunyi Guizhou China

**Keywords:** Alzheimer's‐like pathology, cross‐species analysis, Natural aging, tree shrews, single‐nucleus transcriptomic atlas

## Abstract

The lack of natural aging‐inducing Alzheimer's disease (AD) model presents a significant gap in the current preclinical research. Here, we identified a unique cohort of 10 naturally aging tree shrews (TSs) displaying distinct Alzheimer's‐like pathology (ALP) from a population of 324, thereby establishing a novel model that closely mirrors human AD progression. Using single‐nucleus RNA sequencing, we generated a comprehensive transcriptome atlas, revealing the cellular diversity and gene expression changes underlying AD pathology in aged TSs. Particularly, distinct differentiation trajectories of neural progenitor cells were highly associated with AD pathology. Intriguingly, cross‐species comparisons among humans, TSs, monkeys, and mice highlighted a greater cellular homogeneity of TSs to primates and humans than to mice. Our extended cross‐species analysis by including a direct comparison between human and TS hippocampal tissue under AD conditions uncovered conserved cell types, enriched synaptic biological processes, and elevated excitatory/inhibitory imbalance across species. Cell–cell communication analysis unveiled parallel patterns between AD human and ALP TSs, with both showing reduced interaction strength and quantity across most cell types. Overall, our study provides rich, high‐resolution resources on the cellular and molecular landscape of the ALP TS hippocampus, reinforcing the utility of TSs as a robust model for AD research.

## Graphical abstract

We present a novel Alzheimer's disease (AD) model using naturally aging tree shrews (TSs) that exhibit Alzheimer's‐like pathology (ALP), offering unique insights into human AD progression. Through single‐nucleus RNA sequencing, we mapped the hippocampal transcriptome, revealing key neural progenitor cell differentiation trajectories linked to AD. Cross‐species comparisons demonstrated greater cellular and genetic similarities between TSs and primates than mice, highlighting conserved cell types, synaptic processes, and excitatory/inhibitory imbalances. Cell–cell communication patterns mirrored those in AD human tissue, underscoring the value of TSs as a translational model for AD research.

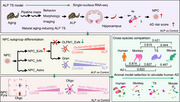

## 1 Introduction

Aging has a profound and devastating effect on the brain, often accompanied by decline in cognitive function and enhanced risk of neurodegenerative disorders like Alzheimer's diseases (AD) [1]. AD manifested by cognition decline closely pertains to the progressive degradation of neurogenesis in hippocampus with age [2]. For many years, researchers have relied upon a very limited number of laboratory intervening models for the investigations of AD, including dogs, mice, and so on [3]. In current studies, investigators tend to construct artificial genetically modified animal models to mimic AD pathogenesis [4]. However, the characteristics of these laboratory animals might be atypical for natural aging‐inducing AD populations, which could inadvertently yield counter‐productive data pertinent to clinical AD patients [5]. Recently, great interest has grown in natural aging‐inducing AD models that are noninvasive and more resembles clinical manifestation [6, 7]. Thus, identifying a novel animal model mimicking natural aging‐inducing AD is of vital importance for clinical AD diagnosis and therapeutics.

Fortunately, tree shrews (TSs; *Tupaia belangeri*), a kind of small animal between primates and insectivora, possessing highly developed motor system and great potentials used for the modeling of human diseases [3], including depression, cognitive disorders, brain ischemia, spinal cord injury, aging, and so on [8, 9, 10, 11, 12]. Previously, studies of TS genomes revealed a closer relation to primates than rodents [13] and reported high homology of AD‐related differentially expressed genes (DEGs) in the brain tissues of TSs with human [13]. In addition to similar genetic, physiological, and neurological characteristics with human, they are also advantageous for small body size, low cost of feeding and maintenance, and short reproductive cycle. More importantly, day‐active TS resembles human circadian rhythms and potentiates as an animal model of human‐like diurnal rhythms [14]. Nevertheless, whether TSs represent ideal models for investigating naturally aging human hippocampus remains largely unknown. In the current context, single‐cell clarification is required to illustrate the high complexity in its cellular composition and dissect the molecular mechanisms underlying hippocampal aging and cognitive decline in natural aging brains. It is of substantial significance to figure out the cell types, cell linage, molecular features, and transcriptional regulation of the aging hippocampus at high resolution.

In the present study, we delineated the distinction of naturally senescent TSs manifesting Alzheimer's‐like pathology (ALP) phenotypes compared with control TSs, establishing a novel model that closely mirrors human AD symptoms in the biomedical research. Besides, single‐nucleus RNA sequencing (snRNA‐seq) assay illustrated the cellular composition, genetic characteristics, and transcriptional regulation of the hippocampus in ALP TSs. Cross‐species analysis further underscored that TSs indeed exhibit great potentials to simulating natural aging‐inducing AD pathology. This high‐resolution resource provides valuable insights into hippocampal cellular and molecular characteristics in ALP TSs, facilitating the identification of potential diagnostic biomarkers and therapeutic targets for AD in the context of natural aging.

## 2 Results

### 2.1 The Successful Verification of Natural Aging‐Inducing AD Phenotypes in TSs

**FIGURE 1:**
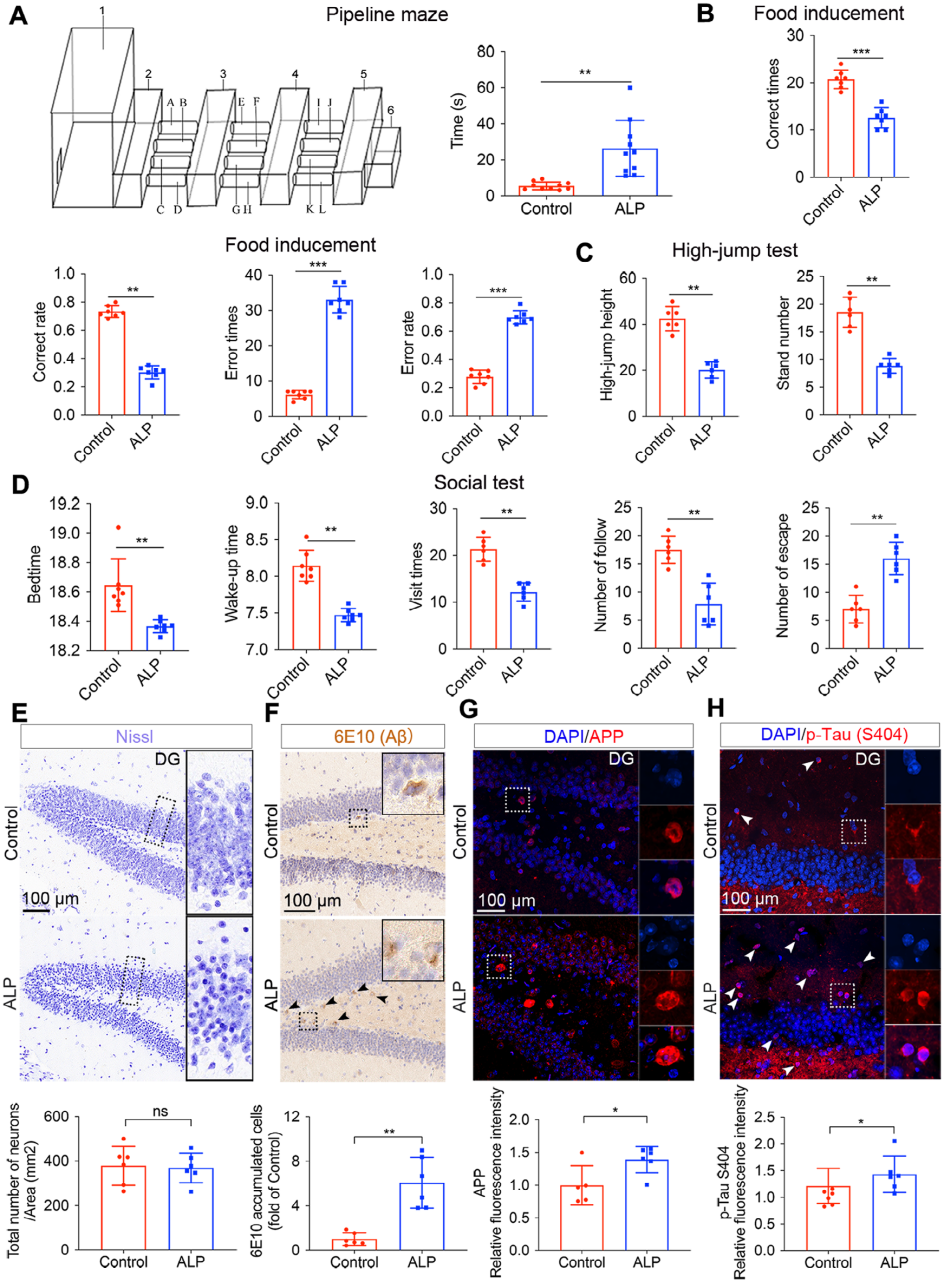
Behavioral and morphological evaluations of natural aging‐inducing AD phenotypes in TSs. (A) Natural aging‐inducing ALP TSs were selected from a cohort of 324 TSs in accordance with the pipeline maze completion time aligned with 90% percentile curve. (B) Comparison of the correct/incorrect times and rates between ALP and control groups in food inducement test. (C) The comparison of high‐jump height and standing times between ALP and control TSs. (D) The waking and sleeping durations, and activity measures (visiting, following and escaping times) within 24 hours between ALP and control TSs. (E) Nissl staining shows pathological changes of hippocampal DG area in ALP and control TSs. (F) Immunohistochemistry and quantification show the Aβ plaques deposition in the hippocampal DG region of ALP and control TSs. (G–H) Immunostaining detects APP and phosphorylated Tau levels in the hippocampal DG region of ALP and control TSs. Scale bar = 100 µm (E, F, G, H). Dots represent the value of quantification for individual subjects (A–D). Individual dots represent the value of quantification for different sections (E–H). The dashed boxes indicate the regions to be magnified. *N* = 5–10/group, independent‐samples *t* test, *p < 0.05, **p < 0.01, ***p < 0.001. ALP, Alzheimer's‐like pathology; APP, Amyloid precursor protein; ns, no significance; p‐Tau, phosphorylated Tau protein; TSs, tree shrews.

In this study, 6 naturally aging TSs with ALP (referred as ALP TSs throughout this article) were selected from a cohort of 324 TSs based on pipeline maze performance (Table S1). These AD phenotypes were further validated through behavioral evaluation, morphological and imaging examination aligned with clinical AD criteria. ALP TSs spent apparent longer time to navigate the pipeline maze compared with control TSs (Figure 1A). Cognitive and motor impairments were evident in ALP TSs, as indicated by lower correct rate and higher error rate in the food‐induced maze (Figure 1B), as well as reduced jump heights and fewer standing attempts in the jumping test (Figure 1C). Social function deficits were also observed, with ALP TSs exhibiting decreased sleep and wake‐up time, fewer visit times and following behaviors, and an increase in escape attempts (Figure 1D). Interestingly, Nissl staining revealed no obvious pathological difference in the hippocampal tissues of ALP TSs compared with control TSs, indicating no overt neural damage in the hippocampus (Figure 1E). However, immunostaining showed augmented Aβ deposition, and elevated levels of amyloid precursor protein (APP) (the source of the Aβ peptide) and phosphorylated Tau—the key components of senile plaques and neurofibrillary tangles, the two histopathological hallmarks of AD [15], in the hippocampus of ALP TSs (Figures 1F–H and S1A,B).

In addition to behavioral and morphological evaluations, magnetic resonance imaging (MRI) [16] was used to detect structural brain changes associated with AD. The T2‐weighted imaging (T2WI), diffusion‐weighted imaging (DWI) images, and 3D reconstructions revealed significant reductions in the volume of white matter, hippocampal size, and hippocampal length in ALP TSs compared with control TSs (Figure S2A–E,G), while the gray matter volume and the lateral ventricle size were significantly increased (Figure S2F–G). Additionally, hippocampal signal intensity in ALP TSs was significantly lower than that of control group (Figure S2H). Finally, the PET–CT reveled significantly lower glucose uptake in the brains of ALP TSs (Figure S2I), further corroborating the presence of AD‐like metabolic dysfunction. All these detections comprehensively demonstrated the successful induction and characterization of AD phenotypes in naturally aging ALP TSs.

### 2.2 Single Cell Transcriptome Profiling of the Hippocampus in Natural Aging‐Inducing ALP TSs

**FIGURE 2:**
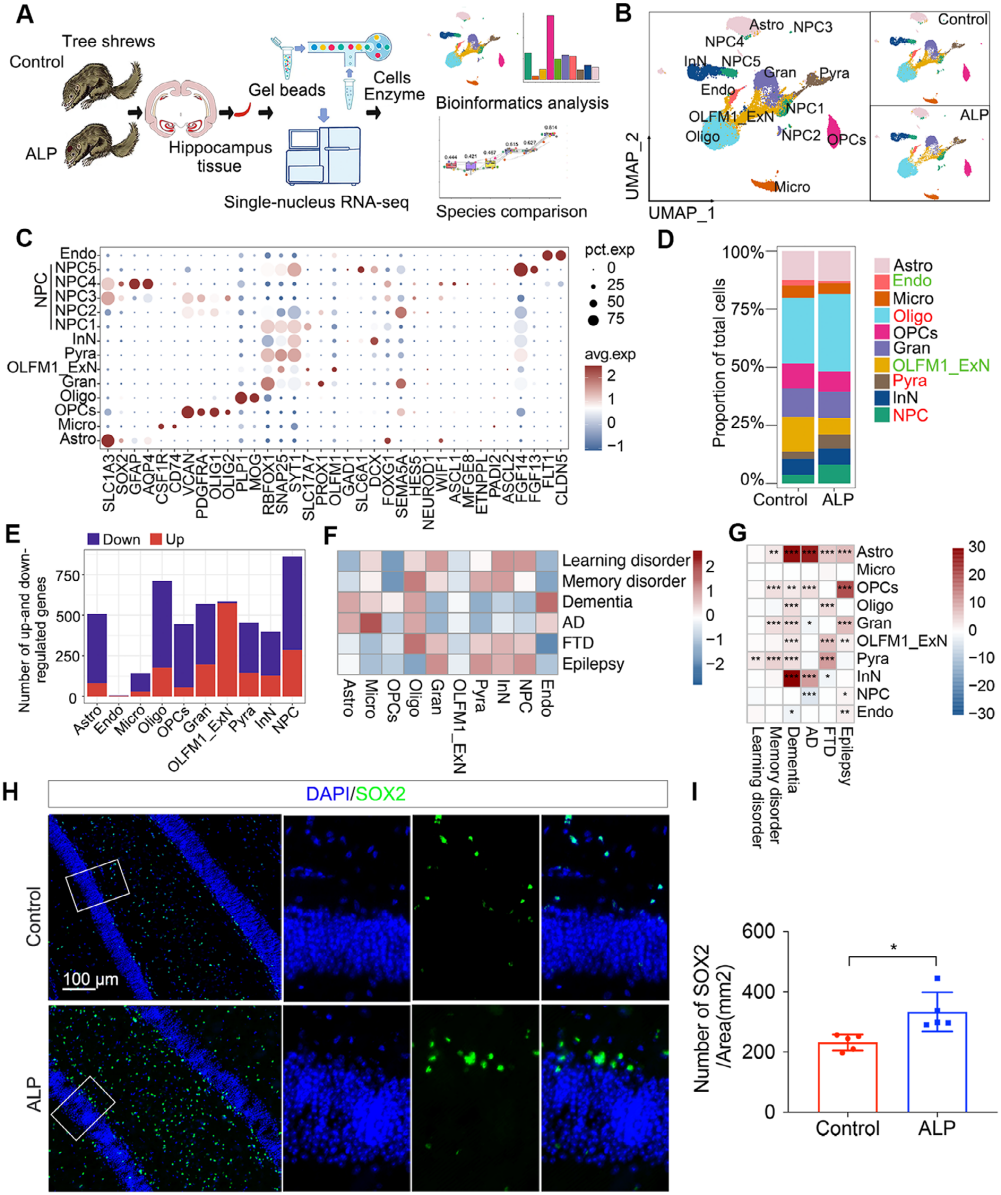
Construction of a snRNA‐seq transcriptomic atlas of natural aging‐induced AD in ALP TSs. (A) The flowchart of snRNA‐seq procedures. (B) UMAP plot depicts 10 major cell populations in the TS hippocampus of ALP and control groups. (C) The dot plot displays the expression of specific marker genes for 10 cell types. (D) The proportion comparison of each cell population within total cells between ALP and control groups, highlighting increases in NPC and Pyra cells and decreases in OLFM1_ExN and Endo cells in ALP TSs. (E) The number of up‐ or downregulated DEGs in each cell populations. (F) The heatmap illustrates the expression patterns of gene sets associated with cognitive impairment across different cell types. The color gradient represents expression abundance, where red indicates higher expression and blue denotes lower expression. (G) The heatmap represents the differential expression changes of gene sets associated with cognitive impairment across distinct cell types. The color scale indicates expression variation, with red signifying increased expression and blue indicating decreased expression. (H) Immunostaining images identify the number of NPC (SOX2^+^) in the hippocampus of ALP and control TSs. Scale bar = 100 µm. (I) Quantification of SOX2^+^ cells between ALP and control groups. Quantification dots represent the value of individual animals. The white boxes indicate the regions to be magnified. *N* = 5/group, independent‐samples *t* test, *p < 0.05. ALP, Alzheimer's‐like pathology; Astro, astrocytes; Endo, endothelial cells; Micro, microglia; Oligo, oligodendrocytes; OPCs, oligodendrocyte progenitor cells; Gran, dentate gyrus (DG) neurons; OLFM1_ExN, a subtype of excitatory neurons; Pyra, pyramidal neurons; InN, inhibitory neurons; NPC, neural progenitor cells; pct.exp., percentage expression; avg.exp., average expression.

TSs are emerging as promising laboratory subjects for neurological research, particularly given the confined availability of primate macaques. However, the cellular and molecular alterations underlying natural aging‐inducing ALP TSs remain unexplored at single‐cell resolution. We conducted snRNA‐seq on hippocampus from 3 ALP TSs and 3 control TSs by using the 10× Genomics platform (Figure 2A), and captured 28,233 high‐quality cells (13,846 from ALP TSs and 14,387 cells from control TSs) (Figure S3A–F) across 10 cell populations: astrocytes (Astro: AQP4), endothelial cells (Endo: FLT1, CLDN5), microglia (Micro: CSF1R, CD74), oligodendrocytes (Oligo: PLP1, MOG), oligodendrocyte progenitor cells (OPCs: Oligo1, Oligo2), dentate gyrus neurons (Gran: PROX1, RBFOX1), OLFM1^+^ excitatory neuron subtype (OLFM1_ExN: OLFM1), pyramidal neurons (Pyra: SNAP25), inhibitory neurons (InN: DCX, SLC6A1, SST, CALB2), and neural progenitor cells (NPC: ASCL1) (Figures 2B,C and S3G–R and Tables S2 and S3). Here, OLFM1‐expressing neurons, defined as a specific subtype of ExN, are implicated in the regulation of axonal growth within both the embryonic and adult central nervous system [17]. By inhibiting RTN4R–LINGO1 interactions, OLFM1 plays a crucial role in preventing axon growth cone collapse, and it may also influence the generation of neural crest cells and modulate olfactory responses [18]. Cell proportion analysis revealed reduced Endo and OLFM1_ExN populations and increased NPC, Oligo, and Pyra populations in ALP TSs (Figure 2D). DEGs were identified for each cell type, the OLFM1_ExN population exhibited a notably higher number of upregulated DEGs compared with downregulated ones (Figure 2E). The volcano plot further illustrated the up or downregulated DEGs in 10 major populations (Figure S4A), and KEGG enrichment analysis highlighted key pathways in different cell types (Figure S4B), including “axon guidance” in NPC, “glutamatergic synapse” in Gran, “oxidative phosphorylation” in OLFM1_ExN, “glutamatergic synapse” in Pyra, “axon guidance” in InN, “neuroactive ligand–receptor interaction” in OPCs, “alanine, aspartate and glutamate metabolism” in Oligo, and so on. Transcription factor (TF) network presented SOX9 and STAT3 potentially driving AD‐related changes like gliosis and inflammation in Astro cells (Figure S4C). These TFs suggest that each cell type may respond to AD pathology through specific transcriptional changes, driving alterations in cellular functions relevant to AD, such as inflammation, neurogenesis, synaptic transmission, and myelination.

We further assessed the expression of gene sets associated with various cognitive impairment, including learning disorder, memory disorder, dementia, AD, frontotemporal dementia (FTD), and epilepsy, across different cell types of ALP TSs (Table S4). Notably, the gene sets related to dementia and AD exhibit higher expression (red) in cell types such as Endo, Astro, Micro, Oligo and OPCs; gene sets related to learning disorder, memory disorder, FTD1, epilepsy show relatively higher expression (red) in NPC, InN, Gran, and Pyra cells (Figure 2F). The observed comparatively lower expression of AD risk genes in neuronal cell types than glial cell types in ALP TS model might be attributed to species‐specific and cell‐type‐specific variations in gene enrichment. Additional association analysis represented significantly differential expression changes of these cognitive impairment‐related gene sets in these cell types of ALP TSs (Figure 2G). This pattern underscores the involvement of neuronal and glial cells in the pathophysiology of these neurological disorders, suggesting their potential roles in disease progression and cellular dysfunction. The hippocampus, a key region affected in AD, supports adult hippocampal neurogenesis (AHN), the lifelong addition of new neurons [19]. AHN persists with mild cognitive impairment and AD but gradually declines as cognitive function deteriorates and AD progresses [20]. Abnormal NPC cell proliferation and self‐renewal are associated with age‐related neurodegeneration and neurodegenerative diseases like AD [21]. Our immunostaining outcomes confirmed elevated SOX2^+^ NPC cells in ALP TS hippocampus (Figure 2H,I), though NPC proliferation remained unchanged (Figure S4D), suggesting abnormal NPC dynamics play a critical role in AD pathology. Subsequent analysis focused NPC transcriptomic changes to further elucidate their contribution to disease progression.

### 2.3 The Differentiation Trajectories of NPC in ALP TSs

**FIGURE 3:**
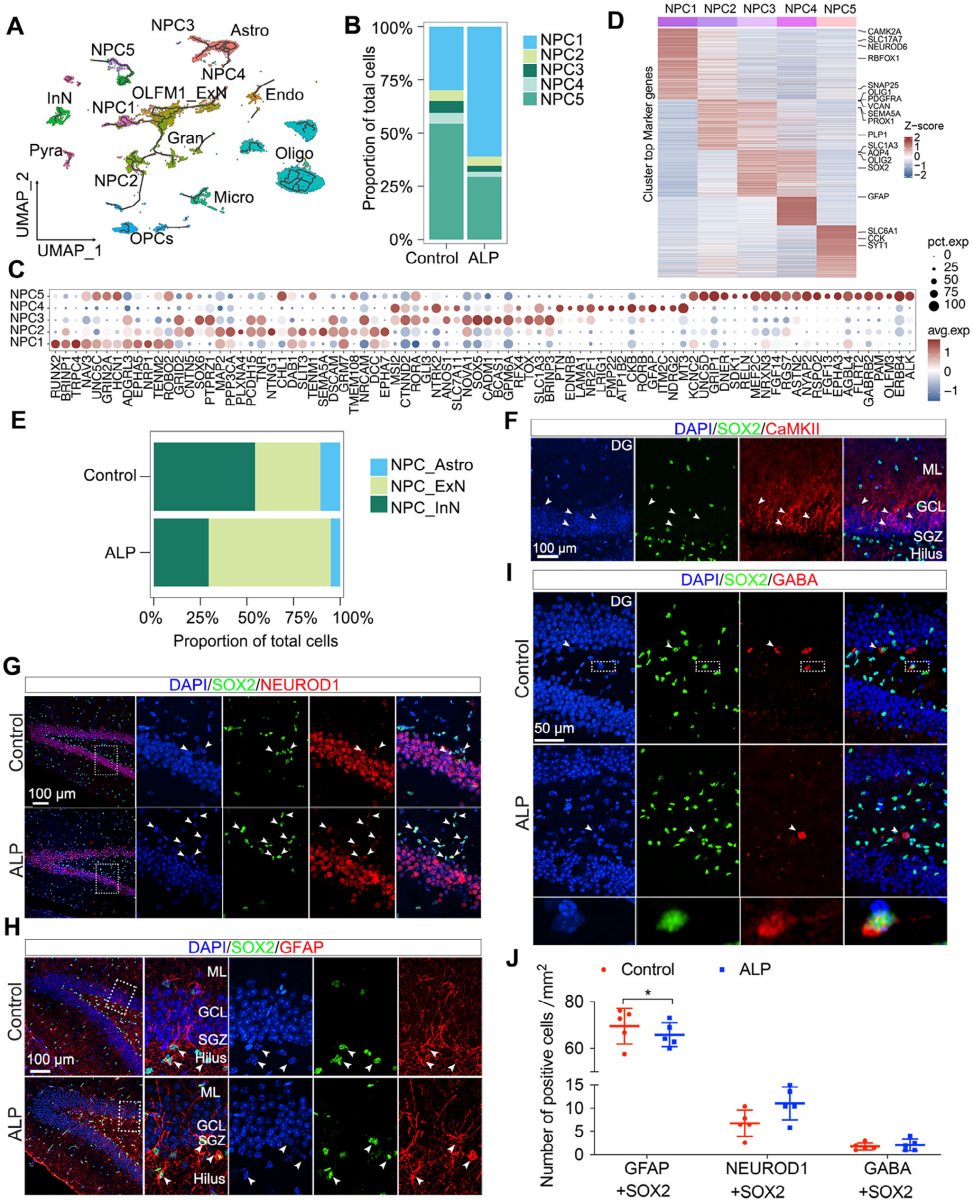
Characteristics of NPC and subpopulations in ALP TSs. (A) The developmental trajectories of five NPC subpopulations with other cell populations analyzed using Monocle3. (B) The proportion comparison of five NPC subpopulations in ALP and control group. (C) The dot plot illustrates the specific expression of neurogenesis and nervous system development‐related genes across the five NPC subtypes. (D) The heatmap shows the specific expression of marker genes across five NPC subpopulations. (E) The proportions of NPC_ExN, NPC_Astro, NPC_InN populations in ALP and control groups. (F) Colocalization identification of Pyra neurons (CAMKII^+^) and NPC cells (SOX2^+^) in ALP and control TS hippocampi. (G–I) Immunostaining identification of NPC_ExN (SOX2^+^Neurod1^+^), NPC_Astro (SOX2^+^GFAP^+^), NPC_InN (SOX2^+^GABA^+^) in TS hippocampi. Scale bar = 100 µm (F–H), 50 µm (I). (J) Quantification of NPC_Astro, NPC_ExN, and NPC_InN cells per unit area (mm^2^) in ALP and control groups. Quantification dots represent the value of individual animals. White dashed boxes indicate the regions to be magnified. White arrows indicate the positive cells. *N* = 5/group, independent‐samples *t* test, *p < 0.05. ALP, Alzheimer's‐like pathology; Astro, astrocytes; Endo, endothelial cells; Micro, microglia; Oligo, oligodendrocytes; OPCs, oligodendrocyte progenitor cells; Gran, dentate gyrus (DG) neurons; OLFM1_ExN, a subtype of excitatory neurons; Pyra, pyramidal neurons; InN, inhibitory neurons; NPC, neural progenitor cells; NPC_ExN, neural progenitor cells with ExN differentiation potential; NPC_Astro, neural progenitor cells with astrocyte differentiation potential; NPC_InN, neural progenitor cells with inhibitory neuron differentiation potential; ML, molecular layer; GCL, granular cell layer; SGZ, subgranular zone; pct.exp., percentage expression; avg.exp., average expression.

To dissect NPC‐specific alterations in the hippocampus ALP TSs, we classified NPCs into five subsets based on expression levels of distinct marker genes: NPC1 and NPC2 expressed CAMK2A; NPC3 and NPC4 expressed AQP4; NPC5 expressed SLC6A1 (Figures 2C and 3A). NPC1, which constituted nearly 70% of NPCs in ALP group, increased compared with control group, while NPC2‐NPC5 exhibited decreasing trends (Figure 3B). Gene Ontology biological process (GO‐BP) enrichment analysis revealed subtype‐specific roles: NPC1 was linked to calcium‐mediated signaling and regulation of synaptic transmission, NPC2 to neuron projection development and cell morphogenesis, NPC3 to cell adhesion and anatomical structure development, NPC4 to neuron projection regulation and sodium homeostasis, and NPC5 to neuronal differentiation and ion transport regulation (Figure S5A–E). These specialized functions support neural regeneration and development. The accompanying dot plot illustrated the distinct expression patterns of neurogenesis and nervous system development‐related genes across the five NPC subtypes (Figure 3C), highlighting the differential roles of these NPC subtypes.

Monocle3 revealed distinct differentiation trajectories of five subpopulations: NPC1/NPC2 progressed toward ExN including Gran and OLFM1_ExN cells, NPC3/NPC4 toward Astro, and NPC5 toward InN (Figure 3A). Marker gene expression demonstrated NPC1/NPC2 expressed ExN‐related genes—NEUROD1, a marker for ExN, and SEMA5A, which is abundantly expressed by developing and adult dentate granule cells [22] (Figure 3D). In the hippocampal DG, Gran cells are ExN as well, sending glutamatergic inputs to other neurons. These indicated that NPC1/NPC2 cells may differentiate into ExN populations. In contrast, NPC3/NPC4 expressed SOX2, a neural precursor marker, as well as Astro markers [23] (AQP4 and GFAP) (Figure 3D). Simultaneous expression of SOX2 and GFAP is characteristic of the type‐1 NPC population, which can be differentiated toward glial lineage (mainly Astro) [24, 25]. These suggested NPC3/NPC4 may differentiate into Astro. NPC5 expressed InN markers (DCX, LHX6, CCK) (Figure 3D), indicating that these cells may differentiate into InN. Therefore, we redefined NPC1/NPC2 as NPC_ExN (neurogenic progenitors), NPC3/NPC4 as NPC_Astro (Astro progenitors), and NPC5 as NPC_InN (InN progenitos). NPC_ExN clustered near ExN subtypes (OLFM1_ExN and Gran cells), NPC_Astro near Astro cells, and NPC_InN near InN, reflecting distinct differentiation pathways (Figure 3D).

Interestingly, in ALP TSs, NPC_ExN increased while NPC_InN and NPC_Astro reduced relative to the controls (Figure 3E), suggesting altered NPC dynamics under ALP condition. Despite the increase in NPC_ExN, the differentiation into the OLFM1_ExN cells was impaired in ALP group, potentially owing to the retention at progenitor stages. Moreover, immunostaining outcomes confirmed no colocalization between Pyra and NPC cells in both ALP and control TS hippocampus, while an increase of NPC_ExN and substantial decrease of NPC_Astro was observed in ALP hippocampus, with NPC_ExN cells localized to DG region (Figures 3F–J and S5F‐H). These findings highlighted NPC subtype diversity and their altered differentiation trajectories in contributing to AD progression, particularly through expanded NPC_ExN populations with impaired terminal differentiation.

### 2.4 Decrease of OLFM1_ExN Differentiated From NPC_ExN Drove AD Progression in ALP TSs

**FIGURE 4:**
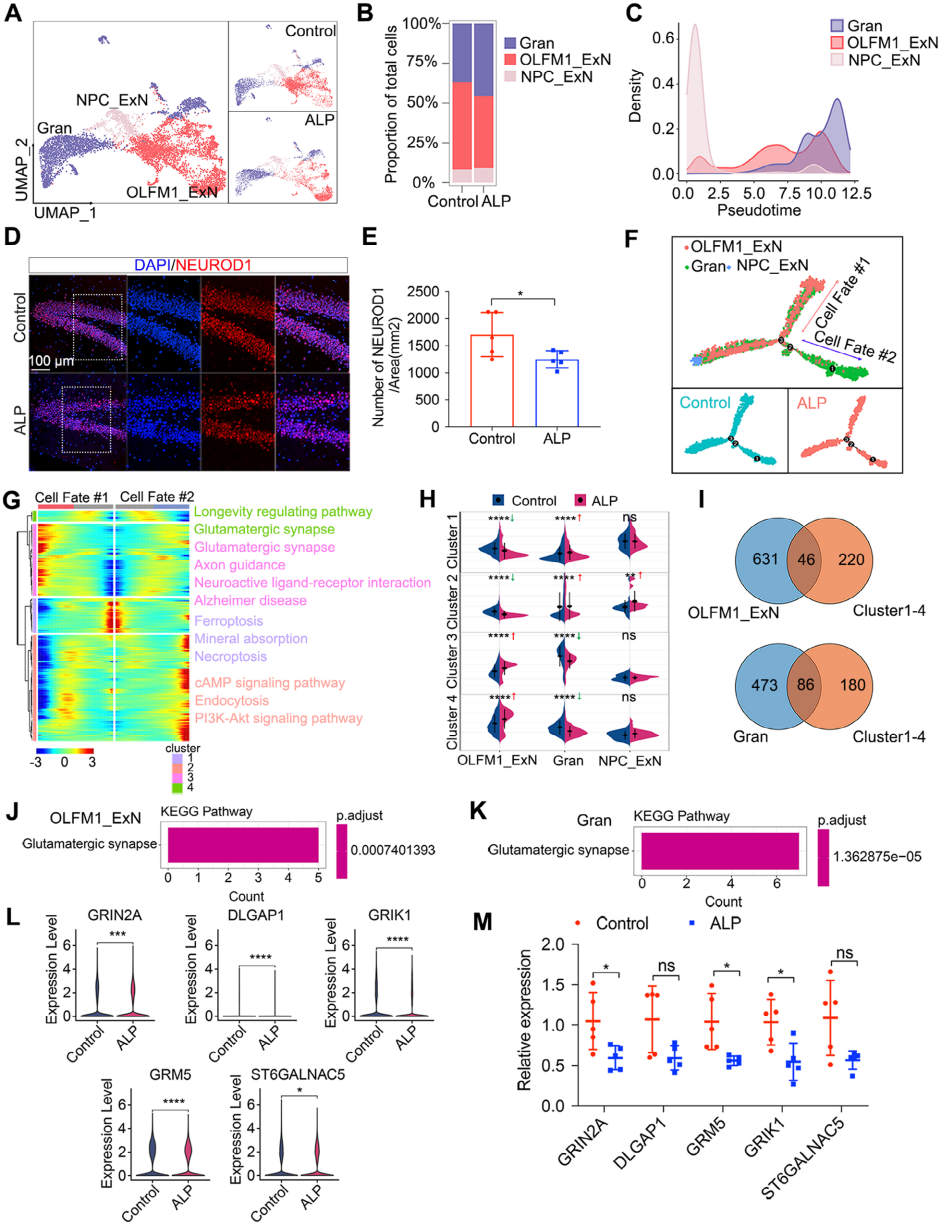
Characteristic profiling of NPC_ExN subpopulations differentiation into OLFM1_ExN and Gran populations. (A) UMAP displays NPC_ExN, OLFM1_ExN, and Gran cell populations. (B) The proportions of three cell populations in ALP and control groups. (C) Pseudo‐time analysis on NPC_ExN, OLFM1_ExN, and Gran cell populations. (D and E) Immunostaining identification and quantification of OLFM1_ExN (NEUROD1^+^) in TS hippocampus of ALP and control groups. Scale bar = 100 µm. Quantification dots represent different sections. *N* = 5/group, independent‐samples *t* test, *p < 0.05. (F) Pseudo‐time analysis on NPC_ExN, OLFM1_ExN, and Gran cell populations. (G) The expression levels of DEGs along the trajectories. DEGs were divided into four clusters with pathways enrichment shown. (H) The violin plot shows enrichment of each gene set across three cell subtypes. Red arrows indicate upregulated enrichment. Green arrows indicate downregulated enrichment. **p < 0.01, ****p < 0.0001. ns, no significance. (I) Venn diagram shows the gene intersection between DEGs in four clusters and AD‐related DEGs in OLFM1_ExN and Gran populations. (J and K) KEGG pathways analysis of upregulated DEGs in OLFM1_ExN population and downregulated DEGs in Gran population. (L) The expression of downregulated DEGs in Glutamatergic synapse pathway. *p < 0.05, ****p < 0.0001. (M) The mRNA expression levels of DLGAP1, GRIK1, GRM5, ST6GALNAC5, and GRIN2A validated by PCR. Quantification dots represent different animals. *N* = 5/group, independent‐samples *t* test, *p < 0.05. ALP, Alzheimer's‐like pathology; Gran, dentate gyrus (DG) neurons; KEGG, Kyoto Encyclopedia of Genes and Genomes; NPC_ExN, neural progenitor cells with ExN differentiation potential; ns, no significance; OLFM1_ExN, a subtype of excitatory neurons.

To explore the role of NPC_ExN differentiation into OLFM1_ExN and Gran populations in AD pathogenesis and progression, we reclustered NPC_ExN, OLFM1_ExN and Gran cells for in‐depth analysis, revealing differences in OLFM1_ExN and Gran proportions between ALP and control groups (Figure 4A,B). Pseudo‐time analysis demonstrated a reduction in NPC_ExN cells and an increase in OLFM1_ExN and Gran populations over time (Figure 4C), with immunostaining confirming fewer OLFM1_ExN neurons in ALP TSs (Figure 4D,E). To understand AD‐related changes along the differentiation trajectories, we analyzed pseudo‐time DEGs across the three cell subtypes (Figure 4F,G). KEGG enrichment analysis highlighted AD‐related pathway dysregulation, with OLFM1_ExN showing downregulation in “ferroptosis,” “mineral absorption,” “cAMP signaling pathway,” and “endocytosis,” while these pathways were upregulated in Gran cells. Conversely, OLFM1_ExN upregulated pathways like “glutamatergic synapse” and “axon guidance,” which were downregulated in Gran cells (Figure 4G,H).

Further analysis identified 46 DEGs after intersecting DEGs in the OLFM1_ExN with overall DEGs across pseudo‐time DEG clusters, and 86 DEGs after intersecting DEGs in the Gran with overall DEGs across pseudo‐time DEG clusters (Figure 4I). KEGG analysis evidenced the enrichment of the “Glutamatergic synapse” pathway in both OLFM1_ExN and Gran populations (Figure 4J,K). AD pathogenesis is highly correlated with the loss of glutamatergic synapses [26, 27]. Key downregulated genes in “glutamatergic synapse” pathway such as DLGAP1, GRIK1, GRM5, ST6GALNAC5, and GRIN2A were validated by real‐time quantitative PCR (RT‐qPCR), which confirmed the downregulation of these genes in the hippocampus of ALP TSs compared with control TSs (Figure 4L,M), consistent with our transcriptomic findings. These findings underscore OLFM1_ExN's critical role in AD progression, where its reduction exacerbates cognitive decline by disrupting glutamatergic signaling.

### 2.5 Cellular and Molecular Characteristics of InN Cells in ALP TSs

Abnormal accumulation of Aβ alters InN cell properties, impairing network activity in AD [28]. To investigate InN heterogeneity, we reclustered InN cells and NPC_InN cells into four major subpopulations based on literature‐defined maker genes [23]: CGE1, CGE2, MGE, and NPC_InN (Figure S6A,B). ALP TSs showed increased MGE and NPC_InN proportions but reduced CGE1 and CGE2 proportions compared with controls (Figure S6C). Monocle3 trajectory analysis indicated that CGE1 and CGE2 progressed from NPC_InN, while CGE2 progressed toward MGE cells (Figure S6D,E). Further pseudo‐time DEG analysis revealed that pathways such as “circadian entrainment,” “glutamatergic synapse,” “axon guidance,” and “giycossphingolipid biosynthesis‐lacto and neolacto series” were upregulated in CGE1, while “cAMP signaling pathway,” “long‐term depression,” “giycossphingolipid biosynthesis‐lacto and neolacto series,” and “pancreatic secretion” pathways were downregulated in CGE2 (Figure S6F,G), linking these two InN subtypes with AD. Additionally, the overlapped DEGs between DEGs in CGE1/CGE2/MGE and pseudo‐time related DEGs were displayed (Figure S6H). KEGG analysis further revealed that “regulation of lipolysis in adipocytes,” “ferrotosis,” “retrograde endocanbinoid signaling” pathway was upregulated in CGE1, while “arrhythmogenic right ventricular cardiomyopathy,” “Hippo signaling pathway,” and “Adheres junction” pathways were downregulated in CGE2 (Figure S6I), indicating increased inhibitory signals in the process of InN cell differentiation in AD. Among those crucial DEGs, MGLLli1 presented as the crucially upregulated gene in CGE1, while CTNNA3 as the CGE2 critically downregulated genes (Figure S6J). These findings provide insights into InN cell alterations and their roles in AD progression in ALP TSs.

### 2.6 Astrocytic Transcriptomic Diversity in ALP TS Hippocampus

Astro cells exhibited high AD risk scores in the TS hippocampus (Figure 2F,G). Reclustering of Astro and NPC_Astro cells identified four subpopulations: Astro1, Astro2, Astro3, and NPC_Astro, with NPC_Astro reduced and Astro1 increased in ALP TS (Figure S7A–C). Pseudo‐time analysis revealed that Astro1 and Astro3 cells originated from NPC_Astro (Figure S7D,E). Additional pseudo‐time DEG analysis demonstrated that “adherens junction,” “bile secretion,” “circadian entrainment,” “glutamatergic synapse,” “oxytocin signaling pathway,” “dopaminergic synapse,” and “ABC transporter” pathways were significantly downregulated in Astro1 cells (Figure S7F,G), indicating that astrocyte involvement in AD risk pathways through various regulatory mechanisms. We further identified 111 overlapping DEGs by intersecting the downregulated DEGs in Astro1 with pseudo‐time DEGs (Figure S7H). KEGG analysis revealed that in Astro1, “glutamatergic synapse,” “long‐term depression,” “synaptic vesicle cycle,” “dopaminergic synapse,” and “GABAergic synapse” pathway was significantly downregulated (Figure S7I), indicating that synapse‐related pathways were downregulated in the Astro differentiation process in AD. In these downregulated pathways, expression levels of CACNA1A, GRIK2, and SYT1 was reduced in ALP group relative to control group (Figure S7J). These findings illustrate Astro's unique molecular features in ALP TSs, offering insights into AD‐related mechanisms.

### 2.7 Transcriptomic Diversity of Hippocampal Oligo in ALP TSs

**FIGURE 5:**
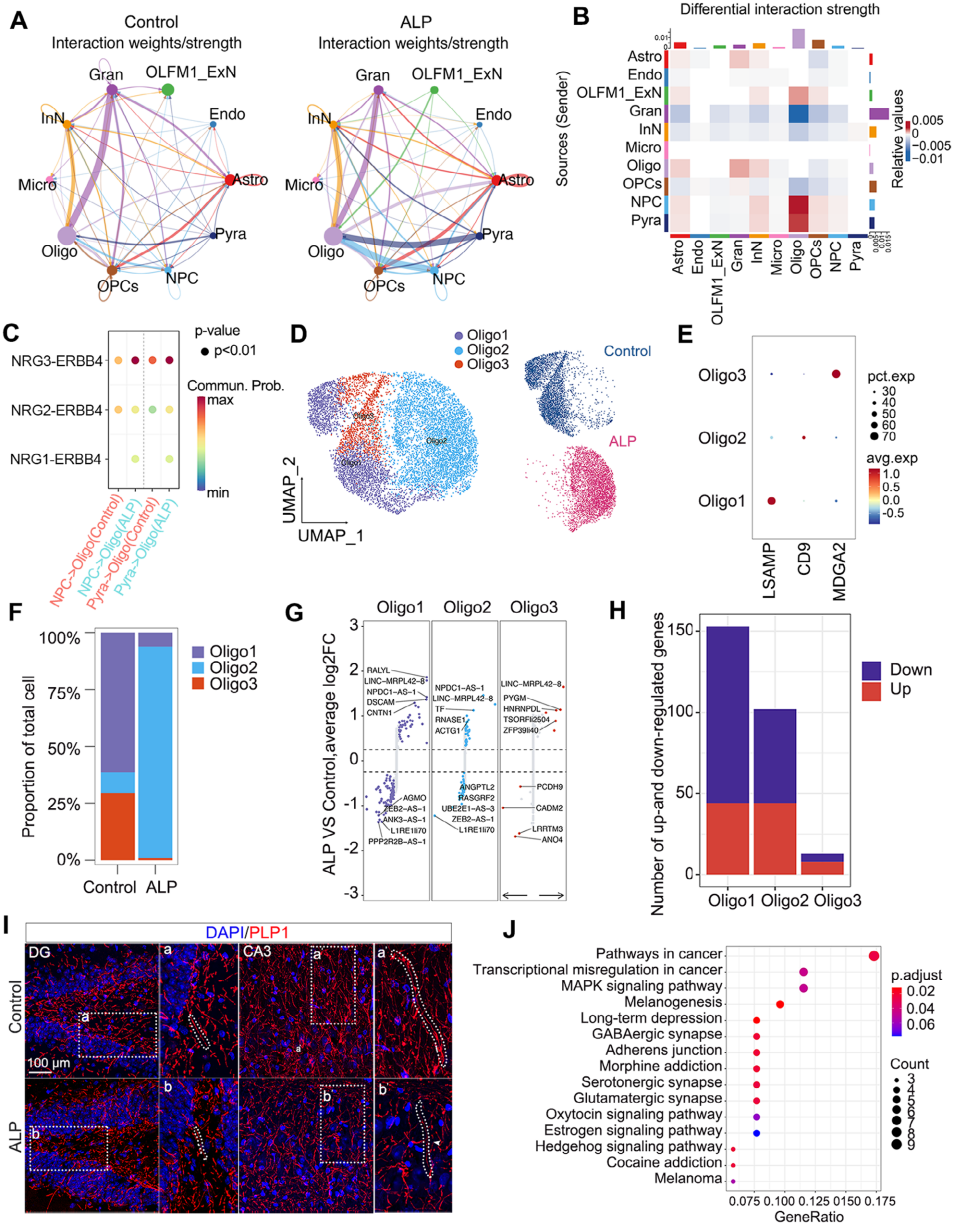
Characteristics of Oligo and their subpopulations in ALP TSs. (A) The interaction intensity map of 10 cell populations. (B) The heatmap displays interaction intensity differences of 10 cell types between ALP and control groups. (C) Dot plot shows the ligand–receptor interaction strength in NPC–Oligo and Pyra–Oligo pairs. The NPC and the Pyra specifically targeted at NGR1–ERBB4, NGR2–ERBB4 pathways of Oligo, and the NGR3–ERBB4 in ligand–receptor pathways was upregulated. (D) UMAP displays the subpopulations of Oligo. (E) The marker gene expression of Oligo subpopulations. (F) The proportions of 3 Oligo subpopulations in ALP and control groups. (G) The volcano plot shows DEGs in 3 Oligo subpopulations. (H) The number of up‐ and downregulated DEGs across 3 Oligo subpopulations. (I) Immunostaining images of PLP1 expression in the TS hippocampus of ALP and control groups. Scale bar = 100 µm. *N* = 5/group. White dashed boxes indicate the regions to be magnified. Irregular dashed lines circle cell morphology of Oligo. (J) KEGG pathways of downregulated DEGs in Oligo2 (ALP group) vs Oligo1 and Oligo3 (control group). ALP, Alzheimer's‐like pathology; Astro, astrocytes; Endo, endothelial cells; Micro, microglia; Oligo, oligodendrocytes; OPCs, oligodendrocyte progenitor cells; Gran, dentate gyrus (DG) neurons; OLFM1_ExN, a subtype of excitatory neurons; Pyra, pyramidal neurons; InN, inhibitory neurons; NPC, neural progenitor cells; Commun.Prob., communications in probability.

Intercellular communication in the hippocampus, crucial for maintaining tissue homeostasis, was examined in ALP TSs using CellChat. Notably, increased interactions were observed between Pyra and Oligo, as well as NPC and Oligo in ALP TSs (Figure 5A). The differential interaction strength heatmap across 10 subpopulations displayed intensified communication signaling between NPC and Oligo populations (Figure 5B). Further ligand–receptor pairs analysis observed a notable increase in the NRG3–ERBB4 signaling between NPC and Oligo populations in AD (Figures 5C and S8A–E). The linkage between NPC and Oligo might play a crucial role in AD pathogenesis. To further explore the heterogeneity of Oligo involved in AD, Oligo cells were reclustered into three subpopulations: Oligo1 (MDGA2), Oligo2 (CD9), and Oligo3 (LSAMP) (Figure 5D,E). ALP TSs exhibited lower proportions of Oligo1 and Oligo3 but higher Oligo2 compared with control TSs (Figure 5F). DEG analysis further highlighted the distinct molecular changes within these subpopulations (Figure 5G), with Oligo1 and Oligo2 showing more DEGs than Oligo3 (Figure 5H). Immunostaining outcomes revealed increasingly disrupted phosphosphingolipids protein (PLP1) expression in the hippocampal CA1, CA2, and CA3 regions of ALP TSs, whereas it remained continuous in the control hippocampus (Figures 5I and S8F). As for KEGG pathways, the downregulated DEGs in ALP Oligo were linked to pathways like “long‐term depression,” “GABAergic synapse,” “serotonergic synapse,” and “glutamatergic synapse” pathways (Figure 5J). Taken together, these findings suggested that intensified intracellular communication between Oligo and NPC might contribute to AD progression, and in particular, the Oligo subpopulations appear to be involved in progressive functional decay of hippocampal cells in ALP TSs.

### 2.8 Cross‐Species Transcriptomic Diversity Highlighted the Potency of TSs as Novel Natural Aging‐Inducing ALP Model

**FIGURE 6:**
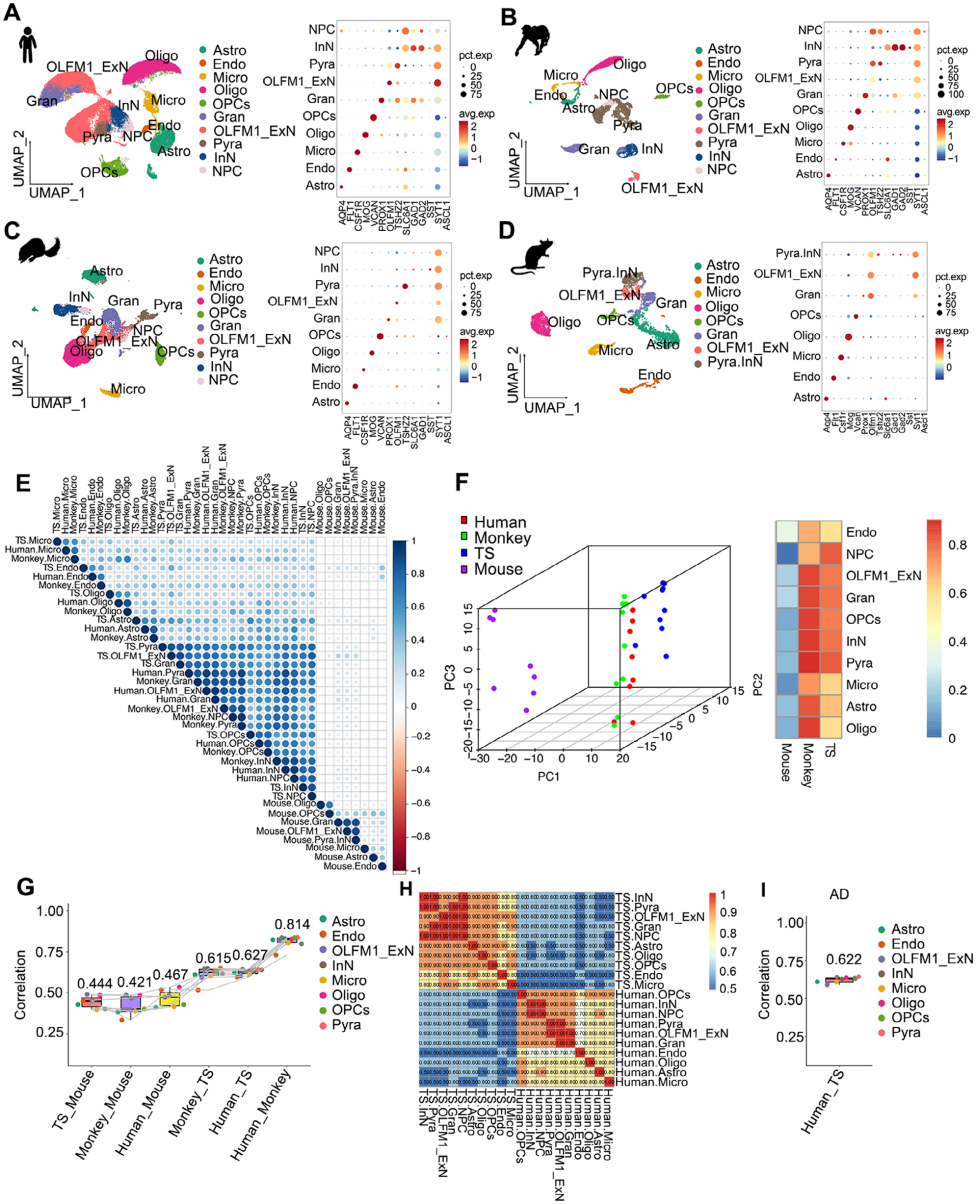
The cross‐species transcriptomic analysis among humans, monkeys, mice, and TSs. (A–D) UMAP visualizes major cell populations in TSs, humans, monkeys, and mice brains. (E) The correlation heatmap of transcriptomic expression in different cell types across species. (F) PCA plot of all cell types for each species, marked by different colors, human (red), monkey (green), TS (blue), mouse (purple). Each dot represents a specific cell type from each species (left). Pearson correlation analysis of all cell types of monkeys, TSs, and mice compared with human (right). (G) The histogram of correlation coefficients among four species. (H) The correlation heatmap of transcriptomic expression in different cell types of ALP TSs and AD humans. (I) The correlation coefficients between ALP TSs and AD humans. ALP, Alzheimer's‐like pathology; Astro, astrocytes; Endo, endothelial cells; Micro, microglia; Oligo, oligodendrocytes; OPCs, oligodendrocyte progenitor cells; Gran, dentate gyrus (DG) neurons; OLFM1_ExN, a subtype of excitatory neurons; Pyra, pyramidal neurons; InN, inhibitory neurons; NPC, neural progenitor cells; pct.exp., percentage expression; avg.exp., average expression.

TS has garnered increasing interest as a promising alternative for modeling natural aging‐inducing AD. We compared hippocampal scRNA‐seq data of TSs, humans, monkeys, and mice to understand cross‐species similarities and differences at the single‐cell level (Figure 6A–D and Table S5). The human hippocampal dataset identified 10 cell subpopulations: Astro, Endo, Micro, Oligo, OPCs, Gran, OLFM1_ExN, Pyra, InN, and NPC (Figure 6A and Table S6). The cell composition identified in TSs and monkeys were highly similar to those in humans, with the same 10 subpopulations present (Figure 6B,C and Table S6). However, in mice, only nine subpopulations were identified, with NPC cells absent from dataset (Figure 6D and Table S6). In addition, the correlation analysis of orthogonal gene expression across species proved that humans and monkeys share the highest genetic similarity, and the transcriptomic profile of TSs was more similar to that of humans than to that of mice (Figure 6E and Table S7). PCA plot and heatmap provide insights into the species‐specific clustering and similarities in terms of cell‐type composition and gene expression in the hippocampus. Humans (red), monkeys (green), and TSs (blue) were clustered relatively close to each other (Figure 6F), suggesting that their hippocampal cell‐type compositions or gene expression profiles are more similar. Also, correlation coefficients confirmed that TSs were more genetically aligned with humans than mice (Figure 6G). Moreover, when comparing the transcriptomic similarities between ALP TSs and AD human, a high degree of genetic consistency and correlation was unveiled (Figure 6H,I). These results suggested that TSs might serve as a suitable model to substitute for humans and monkeys in studies of natural aging‐inducing AD.

### 2.9 Comparative Cellular and Molecular Features of Human and TS Hippocampus in AD

**FIGURE 7:**
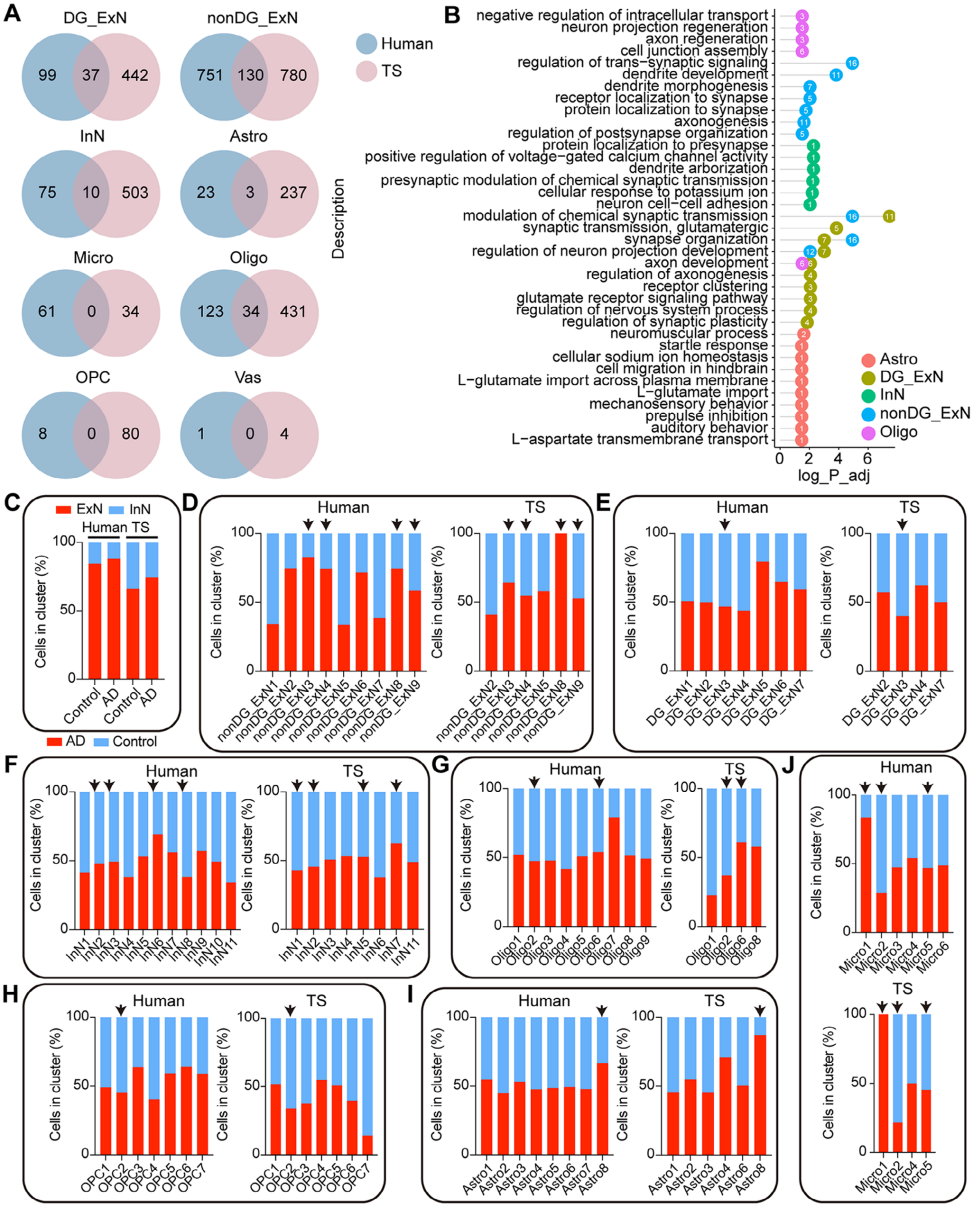
Comparative analysis of DEGs and cell proportion changes in the hippocampus of AD humans and ALP TSs. (A) Overlap of DEGs (FDR < 0.05, |log2FC| > 0.25) across different cell types in the hippocampus of humans and TSs. (B) Biological processes associated with the overlapping DEGs across species, highlighting key functional pathways involved. (C) Changes in the cell proportions of ExN and InN in hippocampal tissues from AD humans and ALP TSs, indicating alterations in ExN/InN balance. (D–J) Proportion changes in different cell subtypes in the hippocampus of AD humans and ALP TSs. Black arrows indicate consistent trends in cell proportion changes between humans and TSs. ALP, Alzheimer's‐like pathology; TSs, tree shrews.

**FIGURE 8:**
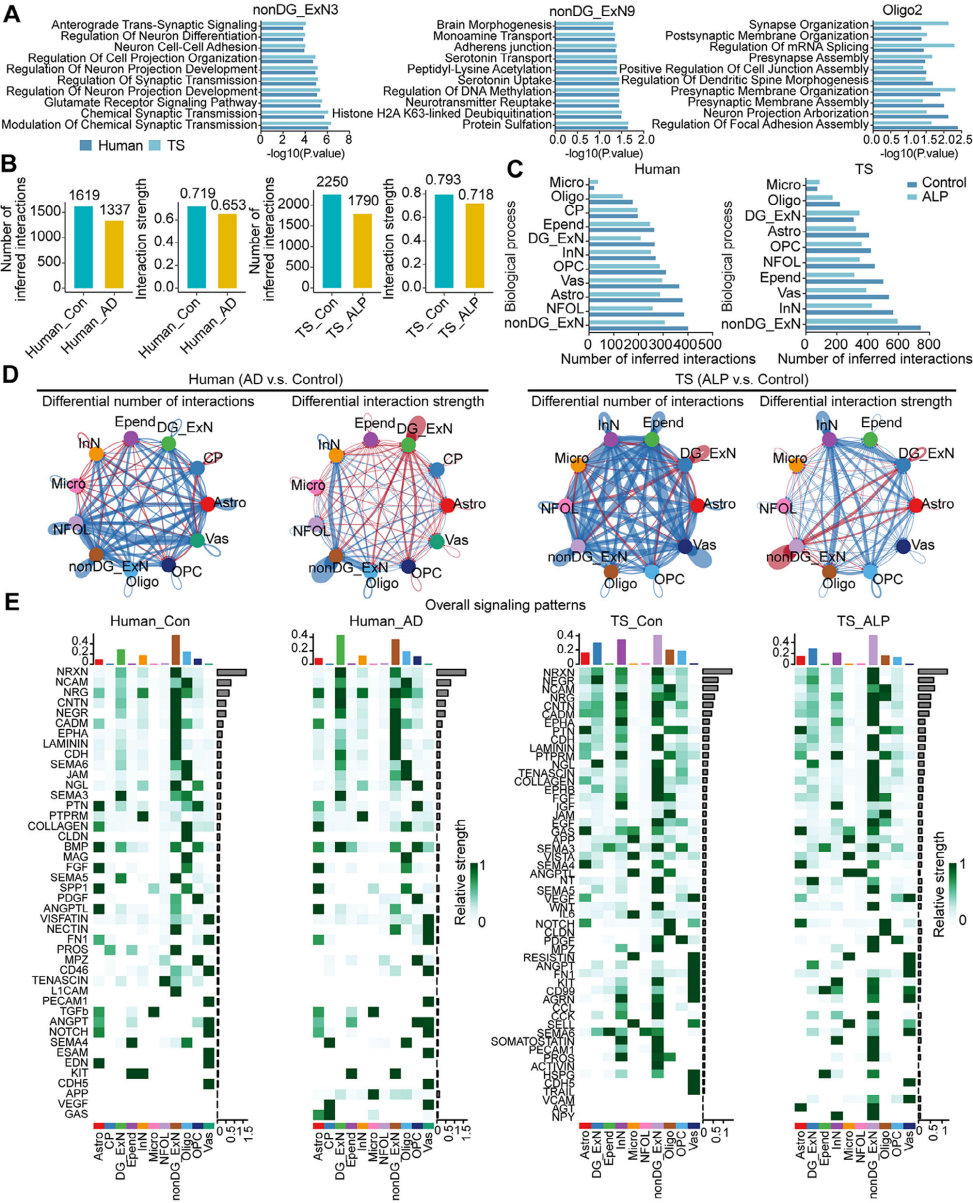
Comparative analysis of differential gene expression, cell communication, and signaling patterns in the hippocampus of AD humans and ALP TSs. (A) Functional enrichment analysis of DEGs in cell subclusters with conserved alteration trends in AD across humans and TSs, highlighting overlapping biological processes. (B) Changes in the overall number and strength of cell–cell communications in the hippocampus of humans and TSs during AD. (C) Variation in the number of cell–cell interactions across different cell types in the hippocampus of AD humans and ALP TSs. (D) Alterations in cell–cell communication patterns among various cell types in the hippocampus of AD humans and ALP TSs. (E) Comparative analysis of cell–cell signaling patterns between different cell types in the hippocampus of humans and TSs in the context of AD. ALP, Alzheimer's‐like pathology; TSs, tree shrews.

To compare the homogeneity and differences between TS and human hippocampal tissue in AD, we performed unified annotation and analysis of cell types in both species to ensure comparability. In the hippocampal tissues of TSs and humans, we identified ten common major cell types. Initially, we analyzed disease‐associated DEGs across different cell types separately (Figure 7A). This analysis revealed species‐conserved DEGs as well as species‐specific DEGs in both human and TS hippocampal tissues. The conserved DEGs were predominantly expressed in nonDG_ExN, DG_ExN, InN, Astro, and Oligo cell types (Figure 7A). These conserved DEGs were enriched in biological processes related to the regulation of chemical synaptic transmission, trans‐synaptic signaling, dendritic spine development, and axon regeneration (Figure 7B), aligning with previous studies that highlight synaptic dysfunction as a core feature of AD and dementia [29, 30, 31, 32]. Imbalances in ExN and InN neuronal activity may lead to large‐scale network dysfunction in the human brain, which contributes to cognitive impairments, as observed in both humans and animal models [33, 34, 35]. Our findings indicated an increased ExN/InN ratio in the hippocampal tissues of AD humans and ALP TSs (Figure 7C). This imbalance was observed not only in altered cell ratios but also in disrupted intercellular communication (described below). To further investigate subtype changes across cell types, we performed graph‐based sub‐clustering analysis on the seven main cell types in the human hippocampus (Figure 7D–J). Using human cell subtypes as a reference, we queried and annotated cell subtypes in TSs. Conserved sub‐clusters were observed, primarily in nonDG_ExN, InN, Oligo, and OPC subpopulations (Figure 7D,F–H). Differential gene expression and functional enrichment analyses on these conserved sub‐clusters in both species revealed consistent biological changes in nonDG_ExN3, nonDG_ExN9, and Oligo2 subtypes, including dysregulation of synaptic transmission and organization, Monamine uptake, and protein modification abnormalities (Figure 8A).

To analyze changes in cell–cell communication in the hippocampus, we conducted CellChat analyses for the primary cell types in both human and TS hippocampal tissues. At an overall level, both the number and strength of cell–cell interactions decreased in the hippocampus of AD humans and ALP TSs (Figure 8B), affecting most cell types (Figure 8C). Notably, the number of Micro‐related interactions significantly increased in both species (Figure 8C), indicating a conserved role of immune imbalance in AD pathogenesis. Additionally, DG_ExN‐related cell communication showed a significant decrease in interaction number but an increase in interaction strength in human AD hippocampus (Figure 8C,D). Conversely, in ALP TS hippocampus, DG_ExN‐related communication showed an increase in interaction number but a decrease in strength (Figure 8C,D). The overall signaling patterns in the hippocampus were similar between humans and TSs, with pathways such as NRXN, NCAM, NRG, CNTN, and NEGR showing the most pronounced expression, which are involved in synaptic function and cognitive resilience [36, 37, 38]. Interestingly, APP and GAS signaling pathways were significantly activated in human AD brain tissue (Figure 8E), consistent with prior reports linking abnormal activation of APP and cGAS to AD pathology and cognitive resilience changes [39, 40, 41, 42]. However, this activation was not prominent in the hippocampus of ALP TSs (Figure 8E). In contrast, VCAM signaling was notably activated in vascular‐associated cells in TSs (Figure 8E). Studies have indicated that VCAM1, a protein that promotes vascular‐immune cell interactions, is significantly elevated in the plasma of elderly individuals and mice, leading to focal upregulation in hippocampal blood endothelial cells of aged mice. Deletion of VCAM1 can counteract the harmful effects of aged plasma on young brains, reversing microglial reactivity and cognitive deficits in aged mice [43].

## 3 Discussion

In this study, a comprehensive single‐nucleus transcriptome atlas of a specific brain region‐hippocampus in the natural aging‐inducing ALP TSs and age‐matched controls was presented. Distinct composition of 10 cell populations included Astro, Endo, Micro, Oligo, OPC, Gran, OLFM1_ExN, Pyra, InN, and NPC in ALP and control TSs, with notable changes observed in NPC, which exhibited significant differentiation potentials into different trajectories closely associated with AD pathological processes. The cross‐species analysis unveiled high similarities between ALP TSs and AD humans, underscoring the feasibility of using TSs as an effective model for simulating natural aging‐inducing Alzheimer's pathology. Together, our study for the first time provides a rich single‐nucleus transcriptomic resource for ALP TS hippocampus, and elicits a solid basis for novel disease model using TSs for its advantages in AD research.

Aging‐induced hippocampal decline leads to profound and inevitable impairment in cognitive abilities and vulnerability to neurodegenerative diseases like AD [44, 45]. Traditional genetically modified animal models like mice, have limitations in simulating natural aging processes. While nonhuman primates closely mimic human AD pathology, their use is constrained by ethical and logistical challenges. Just in search of a natural aging model for human AD conditions, our observations on natural aging TSs revealed that TSs underwent a natural aging process that leads to cognitive decline and neuropathological features resembling human AD, such as amyloid‐beta accumulation and tau pathology. The behavioral characteristics of TSs, including their reliance on complex social behaviors, allow for memory and cognitive testing paradigms that are more translatable to human AD symptoms. Previously, TS has been investigated in the aspects of genome, transcriptome, epigenome, and radiomics, and a basic structural and functional perception closely to human has been established [46, 47, 48, 49, 50]. However, the bulk data are intrinsically restricted in dissecting the heterogeneity and microenvironment of complex tissues. Our study found that TS shares higher transcriptomic similarity with humans and monkeys than with mice. The single‐nucleus transcriptome revealed conserved marker genes across cell types, with TSs showing significant similarities to humans in cellular composition and molecular characteristics. Although monkeys provide high genetic similarity to humans, TS offers practical advantages for longitudinal studies due to their lower maintenance requirements and natural aging‐induced pathology.

The complexity of AD hippocampus necessitates single‐cell‐level investigation to unravel its heterogeneity. Mounting existing sing‐cell resolution studies have provided insights into cell‐type abundance, age‐associated gene expression alterations, and molecular hallmarks of aging in the mouse and monkey hippocampus [51, 52, 53]. In the previous reports [54], the hippocampal cell populations were primarily constituted by neurogenic lineage cells (neural stem cells, transiently amplifying NPC cells, immature neurons, ExN, InN, oligodendrocyte lineage cells, and niche cells), oligodendrocyte lineage cells (OPC and Oligo), niche cells (T cells, Micro, Endo, pericytes, vascular leptomeningeal cells) [53]. Our study complements these findings by establishing a single‐nucleus atlas of TS hippocampus, revealing conserved cellular populations and novel insights into AD‐related alterations. For instance, the proportions of OLFM1_ExN populations were significantly reduced in ALP TSs. AD neurons involved a downregulation of genes associated with synaptic transmission (for example, SNAP25 and RIMS1) and learning [55], consistent with the observed reduction in OLFM1_ExN and Gran cells. These findings highlight impaired neurogenesis and excitatory synaptic signaling as key contributors to AD pathology. Interestingly, the proportions of NPC populations were larger in the hippocampus of ALP TSs. The risk analysis expanded the cell types that correlated with AD development, revealed that Gran, InN, OPCs, NPC, and Pyra had a high correlation with AD, giving a clue to new research direction for clinical treatment. Overall, we have captured a comprehensive landscape of cell types in the natural aging hippocampus of TSs.

Abnormal NPC proliferation and self‐renewal are associated with age‐related neurodegenerative diseases like AD [21]. Decreased NPC proliferation and self‐renewal occur under conditions such as aging, chronic stress, and central nervous system disorders and thereby may contribute to cognitive impairment [56, 57]. In this study, NPCs in ALP TSs showed increased abundance, with differentiation into three subpopulations: NPC_ExN, NPC_InN, and NPC_Astro. Astro played a pivotal role in activating Micro, by releasing NFκB, Ca^2+^, proteoglycans, TGFβ, and complement in mediating neuronal function and AD pathogenesis [58]. Differentiation of NPC into Astro might be entangled in the acceleration of AD progression. Interestingly, NPC_ExN accounted for a high proportion, and two subpopulations were differentiated from NPC_ExN: OLFM1_ExN and Gran, with decreased proportions in ALP TSs and enrichment in glutamatergic synapse pathway. Despite the increase of NPC_ExN in ALP TSs, the differentiation into the OLFM1_ExN cells was decreased in ALP groups, potentially owing to the retention of NPC_ExN without progressing to terminal differentiation. The differentiation into OLFM1_ExN, known for its role in regulating axonal growth and neural development [18], may be indicative of compensatory mechanisms attempting to counteract neurodegenerative processes. Meanwhile, the differentiation into Gran cells, which are implicated in hippocampal function [24, 25], might reflect disruptions in neurogenesis and synaptic integrity associated with cognitive decline in AD. These cellular changes suggest impaired neurogenesis and disrupted excitatory synaptic signaling, contributing to the cognitive and synaptic deficits observed in AD. We further verified glutamatergic‐response‐associated genes in the TS hippocampus, and confirmed the downregulation of DLGAP1, GRIK1, GRM5, ST6GALNAC5, and GRIN2A in the ALP group, confirming that ALP TSs presented weakened glutamate‐related neural signaling. Some reports revealed that the upregulation of glutamatergic‐response‐associated genes in the striatal tissue, enhanced excitatory synaptic transmission in neuronal cultures, and hyperactivity and increased stereotypies in mice [59]. The presence of AD‐related neuropathology contributes to changes in glutamate‐related neural signaling, which subsequently exacerbating an excitatory/inhibitory imbalance and resulting in increased epileptiform activity [60]. This corresponded to the reduction of OLFM1_ExN populations from snRNA‐seq data, and indicated that AD progression might be indispensable with reduction of ExN‐involved glutamatergic signaling. These findings emphasize the potential involvement of altered glutamatergic transmission in AD pathogenesis and highlight NPC_ExN‐derived subpopulations as critical components in understanding disease mechanisms.

Moreover, our analysis reveals a significant overlap in cellular and molecular changes between TSs and humans in the context of AD, underscoring the potential of TSs as a viable animal model for simulating human AD pathology. Both species demonstrated conserved differential gene expression in several key cell types, including nonDG_ExN, DG_ExN, InN, Astro, and Oligo, with these shared DEGs enriched in biological processes related to synaptic transmission, dendritic spine development, and axonal regeneration. These processes are known to play a critical role in AD progression, as synaptic dysfunction and structural abnormalities are hallmark features of AD pathology in humans [61, 62, 63]. Furthermore, our cell–cell communication analysis showed similar trends in overall interaction number and strength across both species’ hippocampal tissues. Specifically, the number of microglia‐related communications was notably increased in both TSs and humans with AD, highlighting a conserved immune imbalance associated with AD. Meanwhile, the overall signaling patterns remained largely conserved, with pathways such as NRXN, NCAM, NRG, CNTN, and NEGR showing significant expression in both species. Interestingly, VCAM signaling was activated in TS vascular cells, which aligns with findings linking VCAM1 to immune response in aging [43]. These findings demonstrate the cellular and molecular consistency between TSs and humans in key aspects of AD pathology, supporting the feasibility of using TS as an animal model for AD. The conserved pathways and cell communication patterns underscore the translational relevance of TSs in studying the underlying mechanisms of AD, potentially enabling the development of therapeutic strategies targeting similar pathological processes in human AD.

Collectively, this study presents a high‐resolution single‐nucleus transcriptomic atlas of the hippocampus in natural aging‐induced ALP TSs, revealing key insights into cellular composition, molecular characteristics, and AD‐related alterations. Notably, the differences in NPC differentiation highlight their importance in AD pathology, suggesting new therapeutic directions. Cross‐species analysis further demonstrated the strong similarity between ALP TSs and human AD, reinforcing TS as a valuable model for studying natural aging‐induced AD. This research establishes TS as a powerful tool for exploring disease mechanisms and potential treatments. However, limitations include the lack of functional validation for key cell types and pathways, and the absence of proteomic or epigenomic data. Additionally, further exploration of sex‐specific differences and cross‐species variations in brain structure is needed to improve the translational value of TS as an AD model.

## 4 Materials and Methods

### 4.1 Animals

A total of 324 TSs aged 5–6 years (140 ± 10 g) were provided by the Animal Center of Kunming Medical University (No. SYXK(Dian)K2020‐0004). Previous published papers defined adult TSs (aged 15–18 months) and aged TSs (aged 6 years or older, ≥72 months) [46, 64]. They were housed in individual dams under a 12‐hour light/dark cycle, with free access to food and water throughout the study. Based on pipeline maze evaluations, 20 TSs were selected and arranged into two groups: ALP group (*n* = 10, TSs with cognitive deficits due to natural senescence) and control group (*n* = 10, age‐matched healthy controls). Of them, 12 TSs (6 per group) were selected for the later behavioral and immunostaining experiments. Detailed information of animals used in this study was provided in Table S1. This study was approved by the Animal Care & Welfare Committee of Kunming Medical University (No. KMMU2020001) and all procedures were carried out in accordance with the Guide for the Care and Use of Laboratory Animals of National Institute of Health.

### 4.2 T2WI and DWI of the TS Brain

TSs were anesthetized with isoflurane, monitored for heart rate, respiratory rate, and temperature, then imaged using a 7.0 T NMR scanner (BioSpec 70/30 USR; Bruker, Germany). EPI scans were performed (TE/TR = 17.766/2000 ms, layer thickness = 1 mm, matrix = 128 × 128, FOV = 3.5 cm × 3.5 cm, volumes = 160). Data were converted from DICOM to nifi format and analyzed with iTK‐SNAP for ROI and volume measurements. 3D reconstructions were done using Surf‐Ice and BrainNet Viewer. T2 images were segmented for gray matter, white matter, and cerebrospinal fluid with SPM12. Apparent diffusion coefficient (ADC) values from DWI were calculated using ParaVision_6.0.1.

### 4.3 Sample Harvest

After imaging, TSs were anesthetized and perfused with 150 mL saline. Brain tissues (cortex and hippocampus) were collected and stored at −80°C for snRNA‐seq and PCR. For immunohistochemistry, Nissl, and immunofluorescent staining, tissues were fixed in 4% paraformaldehyde for 72 h, then dehydrated and embedded in paraffin or frozen. Sections (5 µm for paraffin, 15 µm for frozen) were baked and stored accordingly.

### 4.4 Histological Staining

Histological staining was carried out following established protocols. A detailed description of the staining methods, including the reagents used and the steps involved, is included in Supporting Information. Detailed antibody information is provided in Table S8.

### 4.5 Real‐Time Quantitative PCR

RT‐qPCR was used to verify the differential expression of key genes (DLGAP1, GRIK1, GRM5, ST6GALNAC5, and GRIN2A) identified in transcriptomic analysis. Total RNA was extracted from hippocampal tissue using RNAiso Plus (TaKaRa, Japan) and reverse transcribed into cDNA using RevertAid First Strand cDNA Synthesis Kit (Thermo, USA). PCR amplification was performed using a CFX96 Real‐Time System (Bio‐Rad, USA). The expression levels were normalized to GAPDH using the 2^‐ΔΔCT^ method. Primer sequences are listed in Table S9.

### 4.6 SnRNA‐seq and Data Processing

Hippocampal tissues from three ALP and three control TSs were collected. The reference genome for TS was downloaded (http://ww.treeshrewdb.org/download.html). Raw snRNA‐seq data were processed with CellRanger v.3.1.0, and matrices were analyzed using Seurat (v.4.0). Cells with over 2500 genes, fewer than 200 genes, or more than 5% mitochondrial content were excluded. Data were normalized, scaled, and the top 2000 most variable genes were selected. Principal components were computed, and clustering analysis was performed with a resolution of 2. Uniform Manifold Approximation and Projection (UMAP) was used for visualization [23, 65–67].

### 4.7 Gene Set Score Analysis

Risk genes associated with dementia, AD, learning disorders, memory disorders, FTD, and epilepsy were obtained from the DisGeNET database (https://www.disgenet.org/home/). Human gene sets were mapped to TS genes using homologous gene mapping, including KEGG pathway gene sets. Gene expression scores for these sets were calculated using the “AddModuleScore” function in the Seurat R package. Comparisons of gene expression scores and distributions between the ALP and control groups were performed using the Mann–Whitney *U* test (Wilcoxon rank‐sum test). Genes with statistically significant differences (*p* < 0.05) were identified and reported.

### 4.8 TF Activity Inference

To explore the variation of TF activity between ALP and control groups for TSs, the Dorothea package (https://saezlab.github.io/dorothea/) of R was used to infer TF activity. The top 45 genes with highly variable scores for ALP or control groups (*n* = 90 TFs in total) were displayed for visualization.

### 4.9 Cross‐Species Analysis Among TSs, Humans, Monkeys, and Mice

To perform cross‐species analysis among TSs, human, monkeys (*Macaca mulatta*), and mice, three snRNA‐seq datasets including GSE198323 (human), GSE163737 (monkey), and GSE101901 (mouse) were downloaded from gene expression omnibus (GEO, http://www.ncbi.nlm.nih.gov/geo/). GSE198323 comprises 10 controls and seven AD human, GSE163737 comprises one aging monkey, and GSE101901 contains three mice. Only used the one‐by‐one orthologous genes of TSs (*n* = 21,261), human (*n* = 32,614), monkeys (*n* = 18,908), and mice (*n* = 17,621) were used for cross‐species comparison analysis. Only orthologous genes were used for cross‐species comparison. Data were integrated using Seurat's reciprocal PCA method. Cell‐type clustering and marker gene identification were performed as described above. Cell‐type similarity across species was assessed using the MetaNeighbor package, and Spearman's rank correlations were calculated for gene expression in each subpopulation. PCA and Pearson's correlation analysis were performed using R.

### 4.10 Integration Analysis Between Human and TSs

Human and TS scRNA‐seq data were integrated using Seurat (v4.2.0) [68]. We first integrated human cells (including control and AD brain cells) with TS cells in our companion study. Shared variable genes, selected by “SelectIntegrationFeatures()” function, were used for identifying anchors using “FindIntegrationAnchors().” The two datasets were then integrated together with the “IntegrateData()” function. To visualize all the cells together, we then project the all cells into this UMAP structure of human using “MapQuery()” and “TransferData()” function.

### 4.11 DEG Analysis Using Linear Mixed‐Model Regression

To identify genes differentially expressed in ALP TSs compared with control samples per cell type, *p* values were calculated and FDR‐corrected using Model‐based Analysis of Single‐cell Transcriptomics (MAST). All nuclei from three control and three ALP samples for corresponding cell types were used (Table S2). MAST was used to perform zero‐inflated regression analysis by fitting a linear mixed model. To exclude gene expression changes stemming from confounders, such as sex, unique molecular identifier (UMI), fractions of ribosomal and mitochondrial transcripts, the following model was fit with MAST:

$$\begin{array}{c} \mathrm{zlm}(∼\mathrm{group}+\mathrm{nCount}\_\mathrm{RNA}+\mathrm{percent}.\mathrm{mt}+\mathrm{percent}.\mathrm{rb}\\ +\;\mathrm{Sex},\mathrm{sca},\mathrm{method}=\mathrm{glmer},\mathrm{ebayes}=T) \end{array}$$

where percent.rb is ribosomal RNA fraction and percent.mt is mitochondrial RNA fraction.

### 4.12 Statistical Analysis

The sequencing data were analyzed using R software and Cell Ranger v3.1.0. All statistical analysis was carried out using the SPSS 21.0 software. Spearman correlation analysis was employed to analyze correlation of the transcriptome among humans, monkeys, mice, and TSs. Independent sample *t*‐test was used for comparison between two groups, and the data were expressed as the mean ± standard deviation. ^*^ *p* < 0.05 was considered to be statistically significant.

## Author Contributions

**Ting‐Hua Wang, Liu‐Lin Xiong, and Xiao‐He Tian**: conceptualization and supervision. **Liu‐Lin Xiong, Rong He, and Ruo‐Lan Du**: methodology. **Ruo‐Lan Du, Rui‐Ze Niu, Li‐Ren Huangfu, Yong‐Ping Li, Jing Li, and Lu‐Lu Xue**: investigation. **Li‐Ren Huangfu, Qiu‐Xia Xiao, Lu‐Lu Xue, Chang‐Yin Yu, and Si‐Min Zhang**: visualization. **Ruo‐Lan Du, Rui‐Ze Niu, and Li Chen**: writing – original draft. **Liu‐Lin Xiong, Ting‐Hua Wang, and Xiao‐He Tian**: writing – review and editing. All authors have read and approved the final manuscript.

## Conflicts of Interest

The authors declare no conflicts of interest.

## Ethics Statement

This study was approved by the Animal Care & Welfare Committee of Kunming Medical University (No. KMMU2020001) and all procedures were carried out in accordance with the Guide for the Care and Use of Laboratory Animals of National Institute of Health.

## Supporting information



Supporting Information

Supporting Information

## Data Availability

The raw sequence data reported in this paper have been deposited in the Gene Expression Ominibus of National Center for Biotechnology Information, under accession number GSE235838 at https://www.ncbi.nlm.nih.gov/geo/query/acc.cgi?acc=GSE235838.
