# Supplementary material for: A Single‐Nucleus Transcriptomic Atlas Reveals Cellular and Genetic Characteristics of Alzheimer's‐Like Pathology in Aging Tree Shrews
Source: MedComm (2020). 2025 Mar 15;6(4):e70114. doi: 10.1002/mco2.70114 (PMC11910145; doi:10.1002/mco2.70114)
Supplement: Supplementary file 2 — Supporting Information [file MCO2-6-e70114-s001.pdf]

Supplementary Materials for

A single-nucleus transcriptomic atlas reveals cellular and genetic characteristics of Alzheimer's-like pathology in aging tree shrews

Liu-Lin Xiong *et al.*

*Corresponding author. Email: Ting-Hua Wang: wangtinghua@vip.163.com; Liu-Lin Xiong, liulin.xiong@mymail.unisa.edu.au; Xiao-He Tian, xiaohe.t@wchscu.cn.

This PDF file includes:

Figure S1 to S8
Captions of Table S1 to S9
Materials and methods

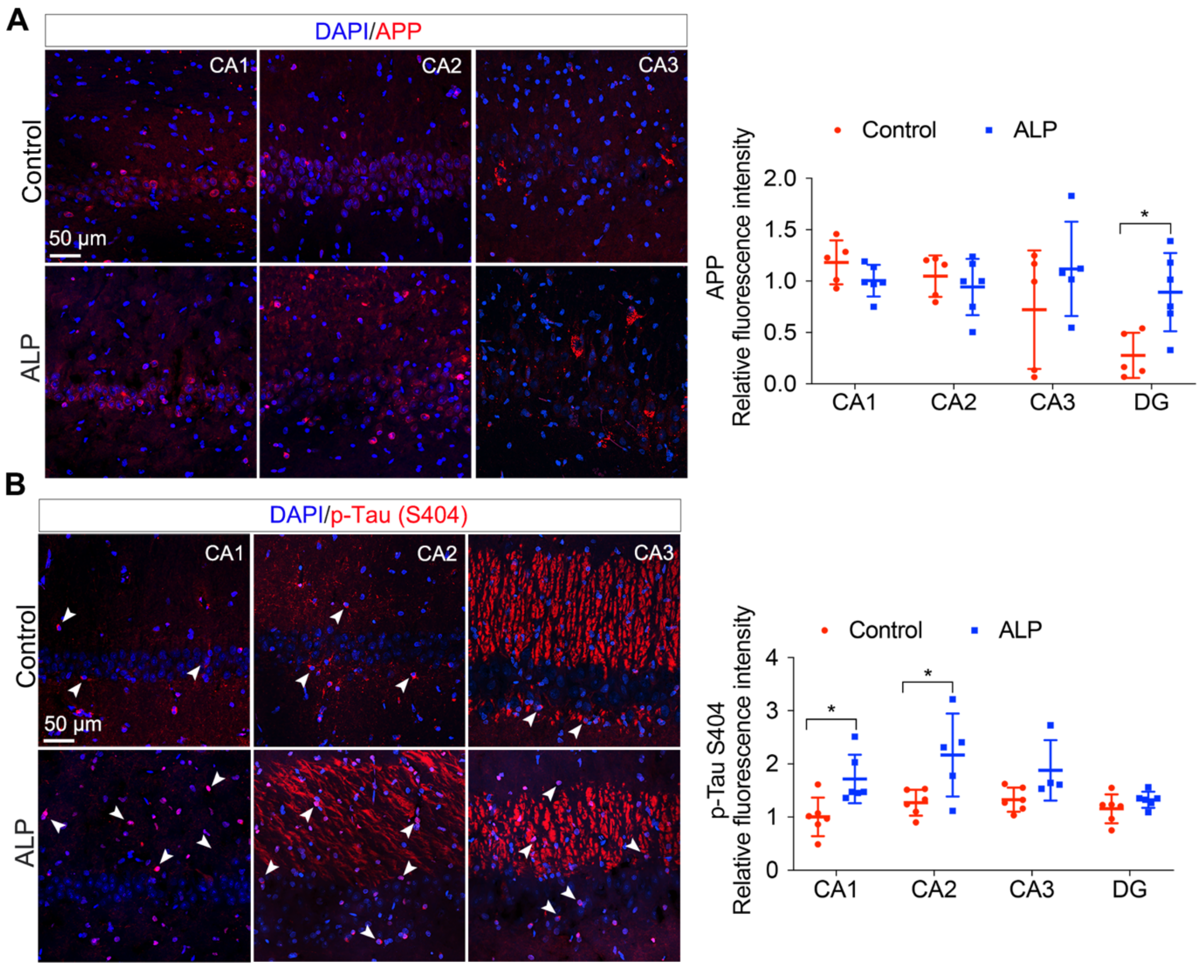

Figure S1 Immunostaining identified the ALP pathology in hippocampal CA areas. (A-B) Immunostaining images shows the levels of APP and phosphorylated Tau in the hippocampal CA areas of ALP and Control TS. Scale bar = 50μm. Quantification of fluorescence intensity of APP and S404 in the hippocampal CA1, CA2, CA3 and DG areas. Dots represent the value of quantification for individual subjects. N = 6/group, independent-samples *t* test, *$p < 0.05$. APP, amyloid precursor protein; p-Tau, phosphorylated Tau; ALP, Alzheimer's-like pathology.

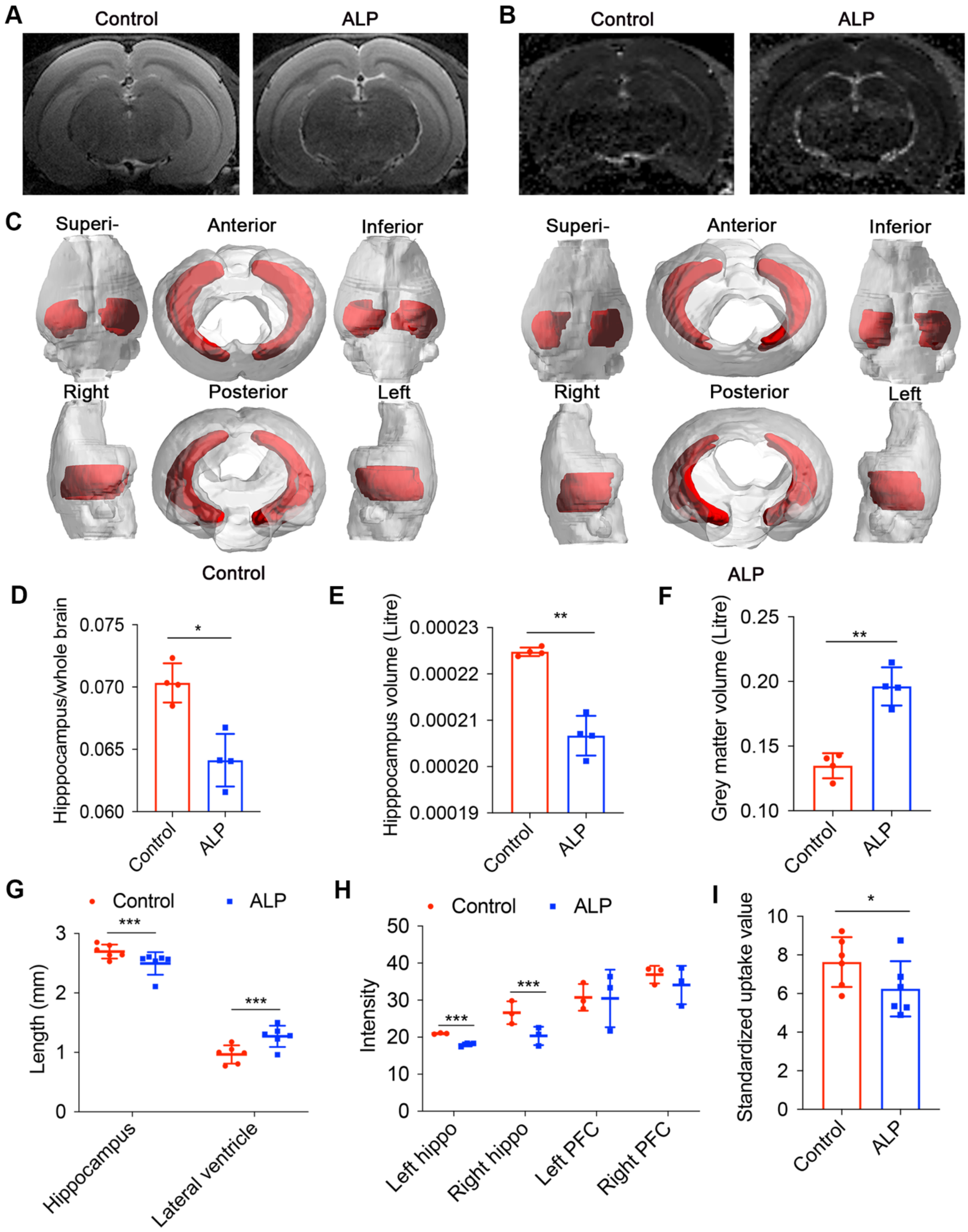

Figure S2 Imaging examination identification of AD pathology in natural aging-inducing ALP TS. (A) The T2-weighted imaging (T2WI) of coronal brain sections between Control and ALP groups. (B) The diffusion-weighted imaging (DWI) of coronal brain sections between

Control and ALP groups. (C) The 3D reconstruction of brains from Control and ALP TS, showing superior, inferior, anterior, posterior, right and left perspectives. Red highlights denote the hippocampus. (D-F) Comparison of white matter volume, hippocampal/whole brain ratio, and grey matter volume between Control and ALP groups. (G) Comparison of hippocampal and lateral cerebral ventricle lengths in ALP and Control groups. (H) The signal intensity measurements of the hippocampus and prefrontal lobe in ALP and Control groups. (I) The standard uptake volume of whole brains in ALP and Control TS detected by PET-CT. Quantification Dots represent the value of individual subjects (D-I). N = 4-6/group, independent-samples *t* test, $^{*}p < 0.05$, $^{**}p < 0.01$, $^{***}p < 0.001$. ALP, Alzheimer's-like pathology; Hippo: hippocampus; PFC: prefrontal lobe.

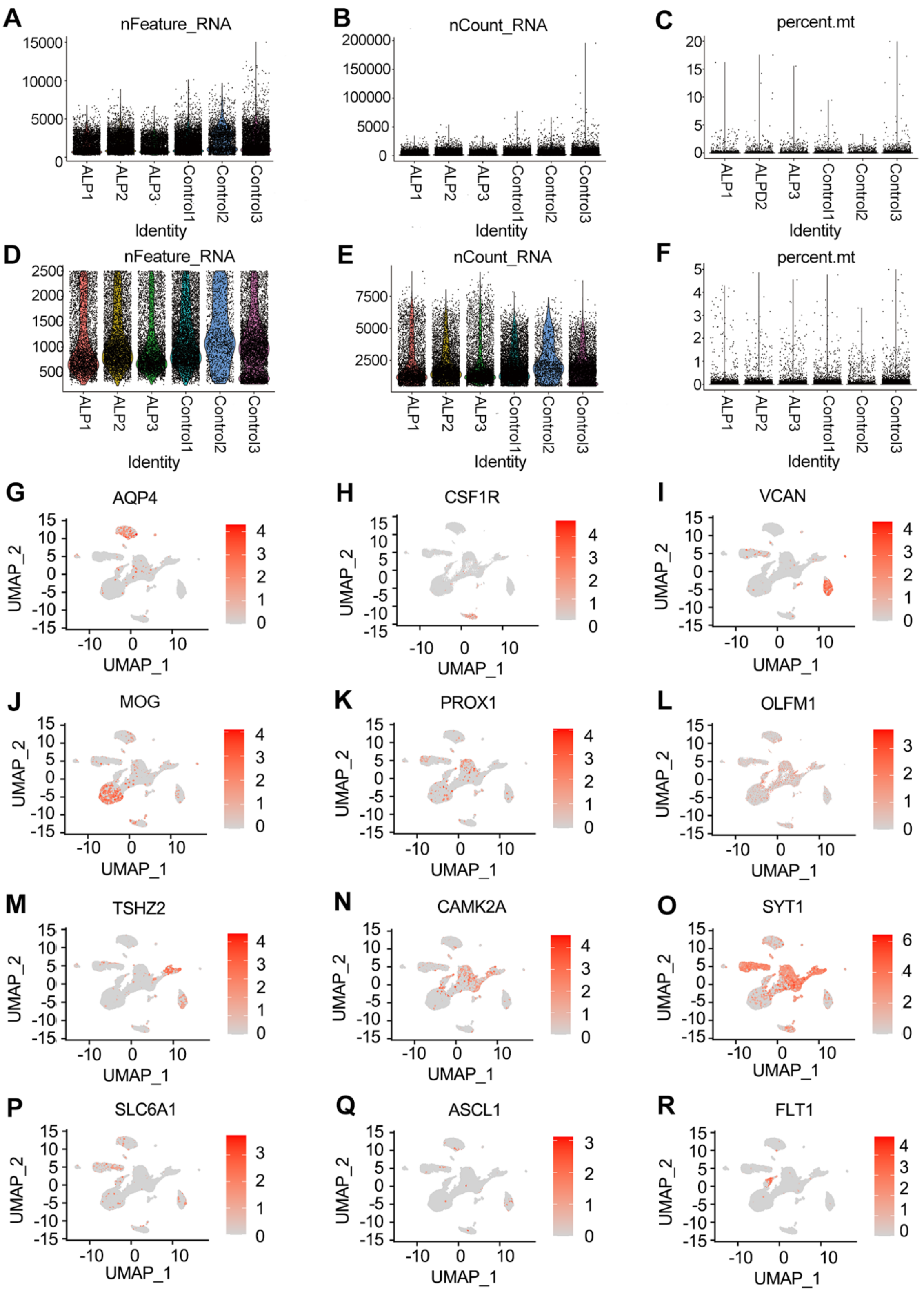

Figure S3 Quality control and marker genes of identified cell populations.

(A) Violin plot for the number of mRNAs for each sample before quality control. (B) Violin plot for the reads of mRNAs for each sample before quality control. (C) Violin plot for the

percentage of mitochondrial genes for each sample before quality control. (D) Violin plot for the number of mRNAs for each sample after quality control. (E) Violin plot for the reads of mRNA for each sample after quality control. (F) Violin plot for the percentage of mitochondrial genes for each sample after quality control. (G-O) UMAP plot for the distribution of specific cell-type marker genes. ALP, Alzheimer's-like pathology; UMAP, uniform manifold approximation and projection.

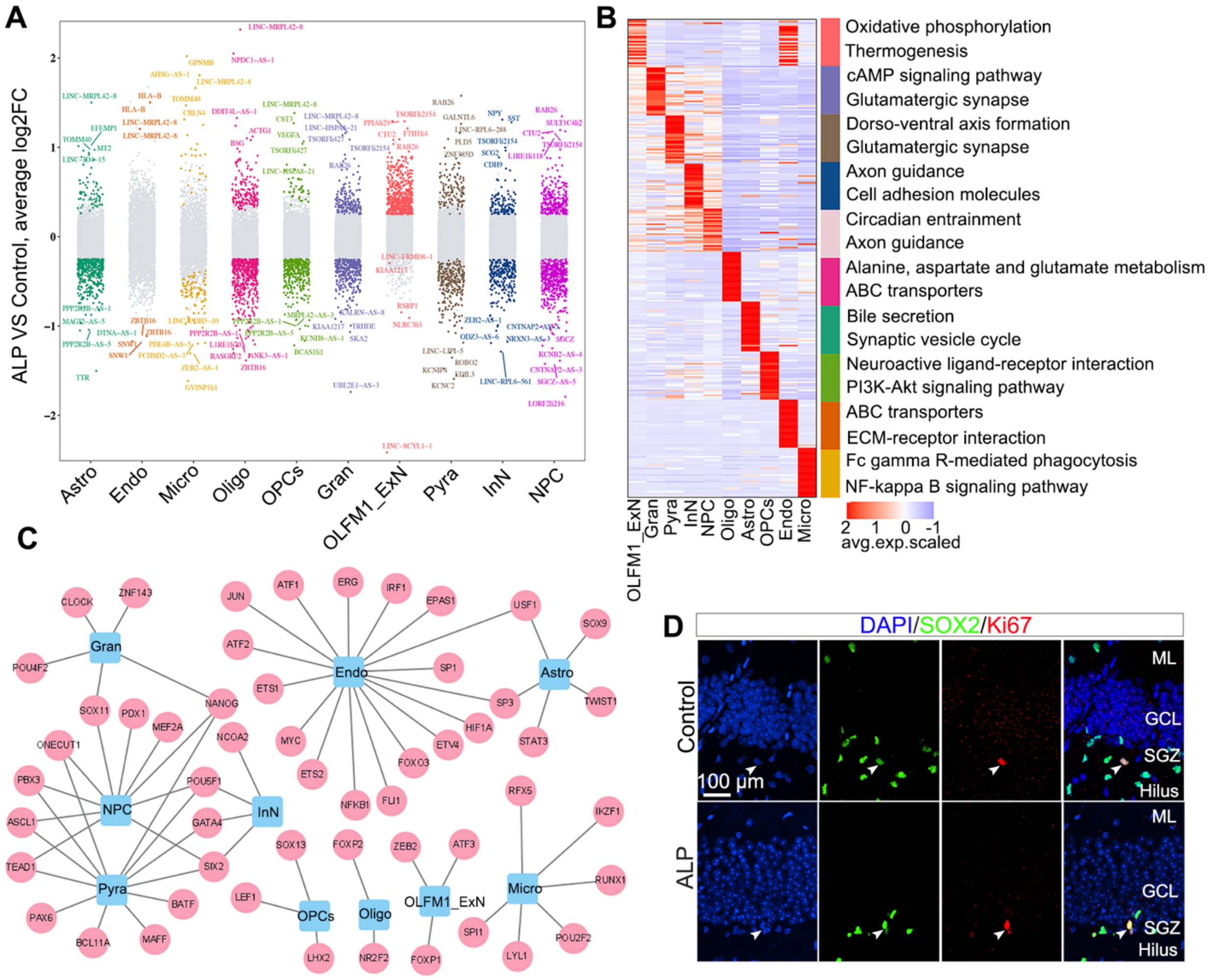

Figure S4 Comparison of cellular differential expression and biological functions between ALP and Control hippocampus.

(A) The volcano map shows DEGs in 10 cell populations, and the most up-regulated and down-regulated 5 genes were marked out. (B) The significant pathways from top 20 terms enriched in 10 cell populations. (C) The interaction network of transcription factors in different cell populations. (D) Immunostaining images identified the proliferation of NPC ($SOX2^{+}Ki67^{+}$) cells in the hippocampus of ALP and Control TS. N = 5/group. Scale bar = 100 μm. White arrows indicate the positive cells. ALP, Alzheimer's-like pathology; Astro: Astrocytes; Endo: endothelial cells; Micro: microglia; Oligo: oligodendrocytes; OPCs: Oligodendrocyte progenitor cells; Gran: dentate gyrus (DG) neurons; OLFM1_ExN: a subtype of excitatory neurons; Pyra: pyramidal neurons; InN: inhibitory neurons; NPC: neural progenitor cells; avg.exp., average expression; ML, molecular layer; GCL, Granular Cell Layer; SGZ, subgranular zone.

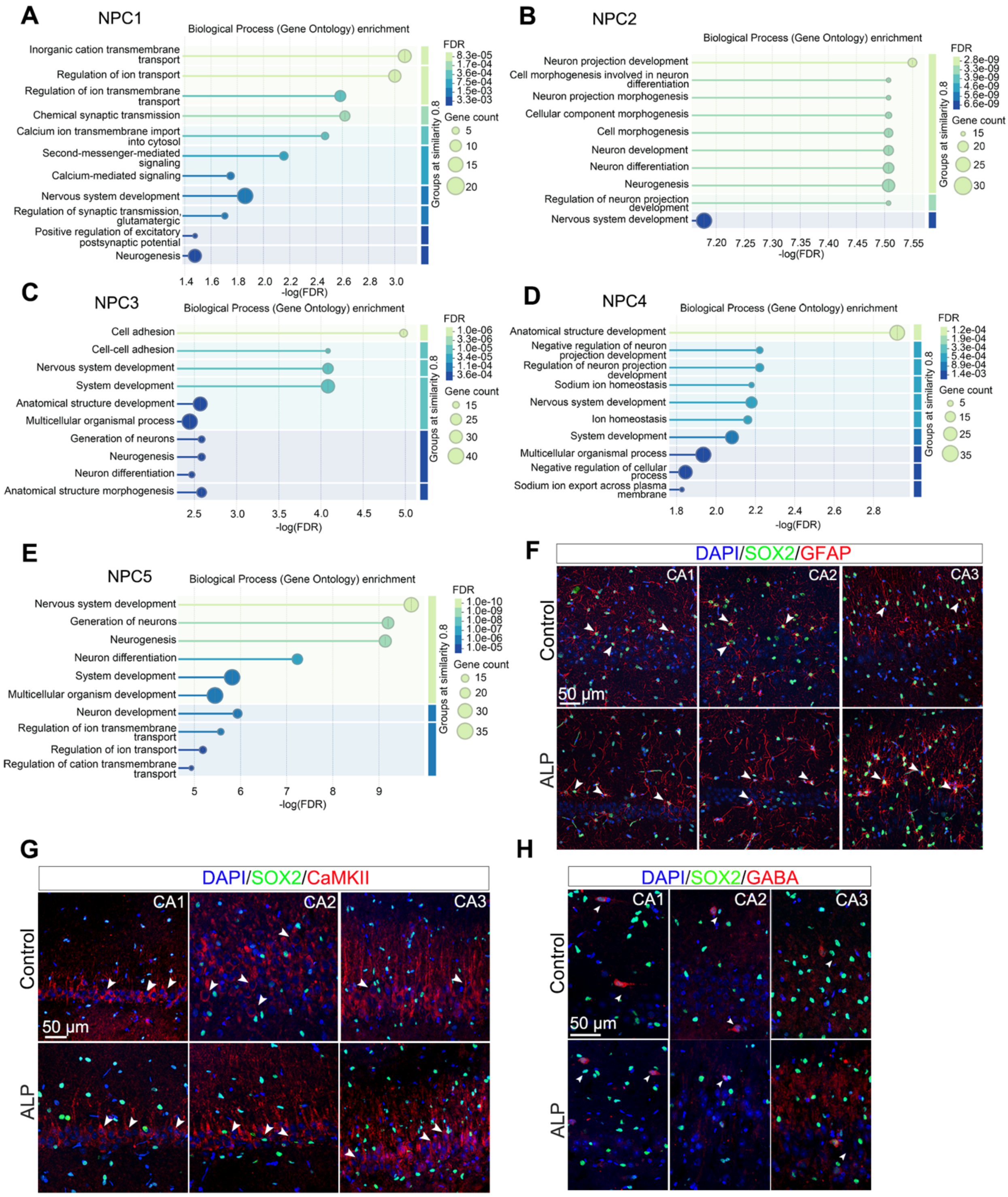

Figure S5 GO enrichment analysis and immunostaining of NPC subpopulations in hippocampus of ALP and Control TS. (A-E) Gene Ontology Biological Process (GO-BP) enrichment analysis for the five subtypes of NPCs between ALP and Control groups. The expression of Pyra and NPC in hippocampal CA1 and CA2 areas of ALP and Control TS. (F-H) The expression of (F) NPC_Astro, (G) Pyra and (H) NPC_InN cells in hippocampal CA1, CA2

and CA3 areas of ALP and Control TS. Scale bar = 50 μm. N = 5/group. White arrows indicate the positive cells; ALP, Alzheimer's-like pathology.

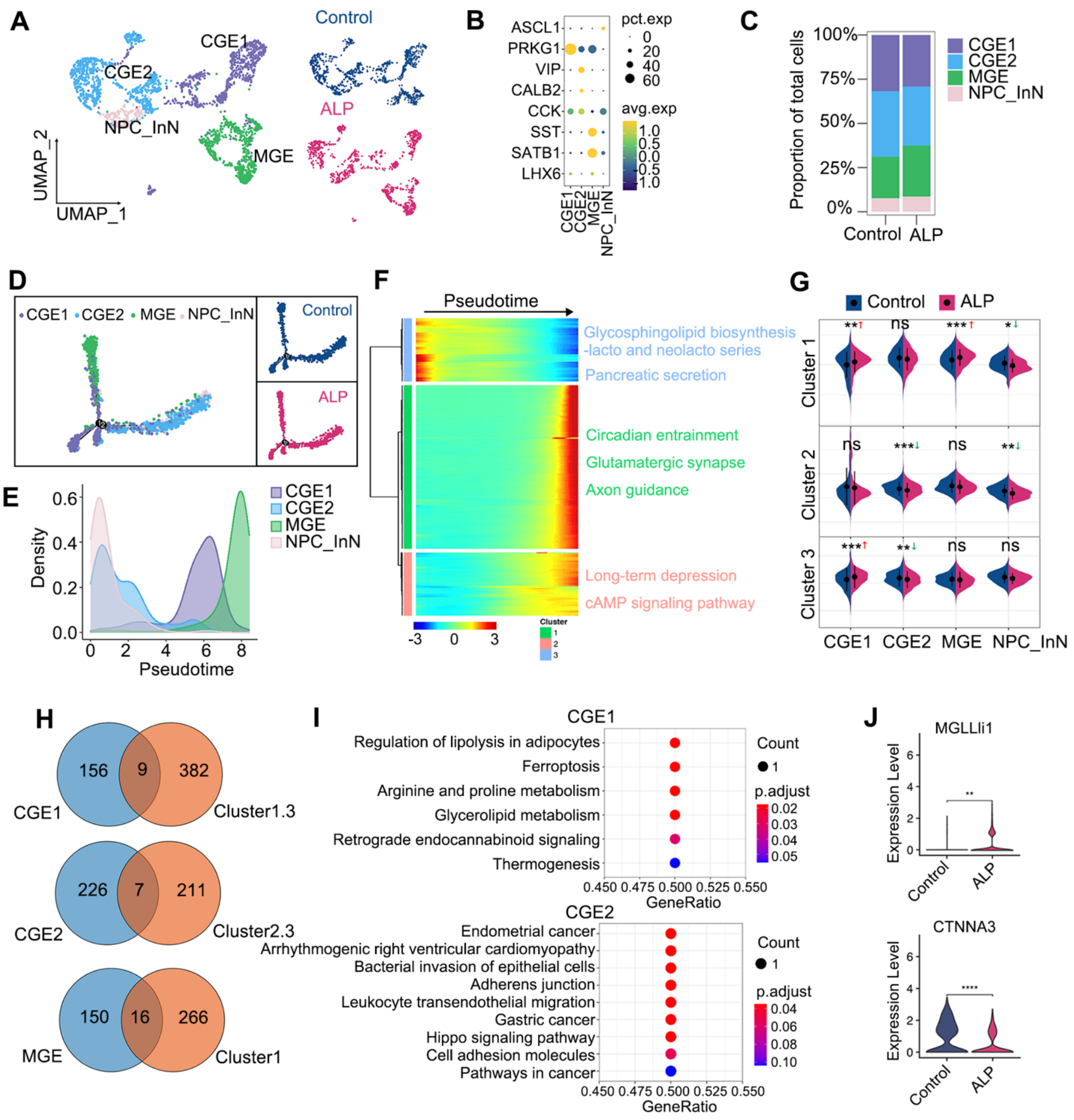

Figure S6 The development trajectory of InN subpopulations.

(A) UMAP plot of NPC_InN and InN cell subpopulation. (B) The marker genes of InN cell subpopulation. (C) The proportions of NPC_InN and three subpopulations in ALP and Control group. (D) Pseudo-time analysis of NPC_InN and InN subpopulations. (E) The proportion changes of each cell populations with time proceeding. (F) The expression level and functional enrichment of pseudo-time DEGs. DEGs were divided into three clusters with their expression mode and enrichment pathways. (G) The violin map showed enrichment comparison of each gene set in different cell types. Red arrows indicate upregulated enrichment. Green arrows indicate downregulated enrichment. (H) The Venn Map showed the genetic intersection between DEGs of

4 clusters and DEGs related to AD in InN cell subpopulation. (I) The KEGG pathways analysis of the up-regulated DEGs in CGE1 and the down-regulated DEGs in CGE1 and MGE. (J) The expression of the up-regulated DEGs in CGE1 and the down-regulated DEGs in CGE1 and MGE. *$p < 0.05$, **$p < 0.01$, ***$p < 0.001$, ****$p < 0.0001$. ALP, Alzheimer's-like pathology; NPC_InN: neural progenitor cells with inhibitory neuron differentiation potential; pct.exp., percentage expression; avg.exp., average expression.

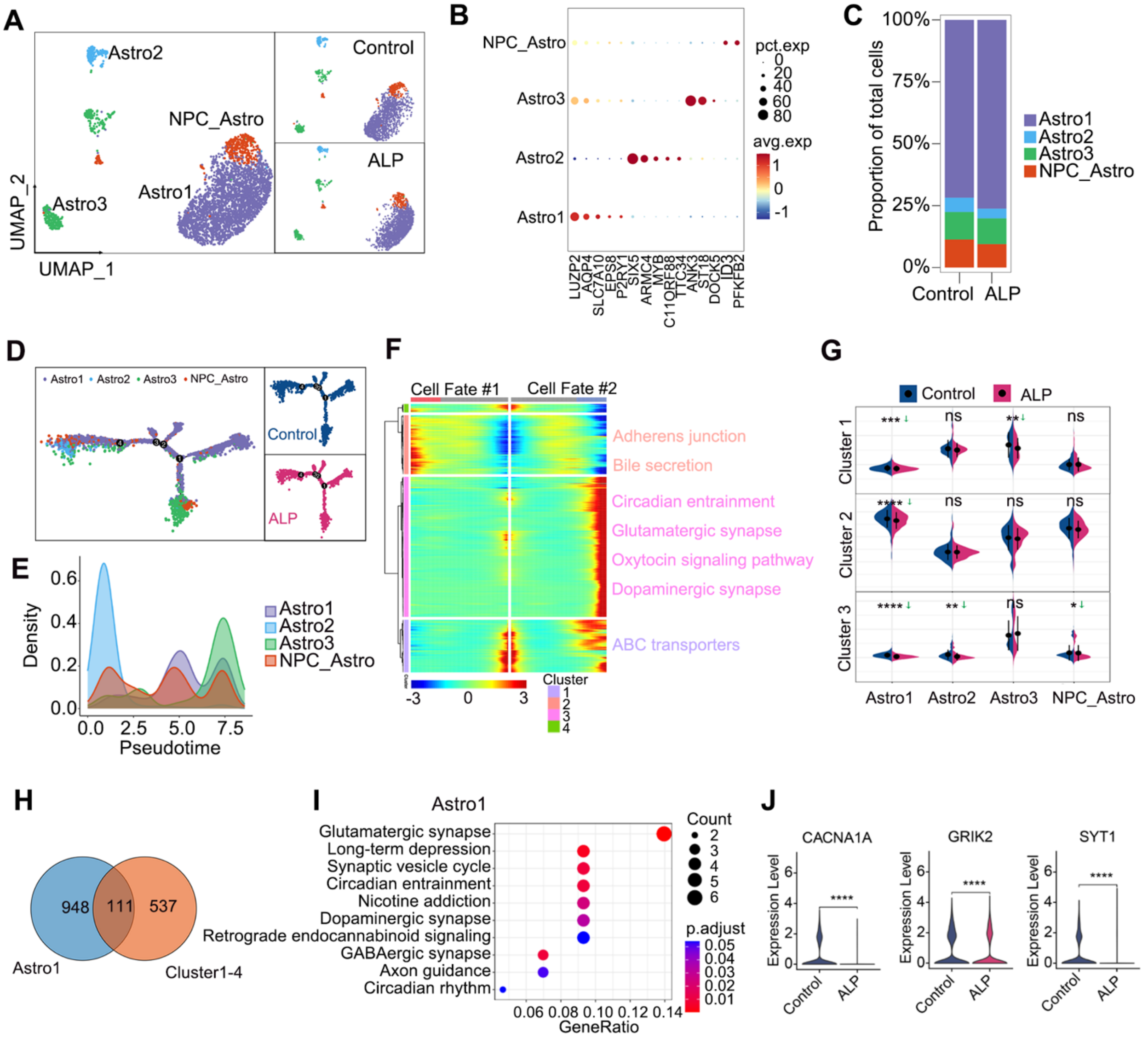

Figure S7 Astro subpopulations characteristics in ALP TS

(A) UMAP plot of NPC_Astro and Astro cell subpopulation. (B) The marker genes of Astro cell subpopulation. (C) The proportion of NPC_Astro and Astro subpopulations in ALP and Control group. (D) Pseudo-time analysis of NPC_Astro and Astro subpopulations. (E) The proportional changes of each cell populations with time. (F) The expression level and functional enrichment of pseudo-time DEGs. The DEGs were divided into four clusters with their expression mode and enrichment pathways. (G) The violin map showed enrichment of each gene set across 4 cell subtypes. Red arrows indicate upregulated enrichment. Green arrows indicate downregulated enrichment. (H) The Venn map shows the gene intersection between pseudo-time DEGs of 4 clusters and DEGs related to AD in Astro1 cell subpopulation. (I) The KEGG pathways of the up-regulated DEGs in Astro1 and the down-regulated DEGs in Astro1. (J) The expression of the up-regulated DEGs in Astro1 and the down-regulated DEGs in Astro1 and Astro3. *$p < 0.05$, **$p < 0.01$ ***$p < 0.001$, ****$p < 0.0001$. NPC_Astro: neural progenitor cells with astrocyte

differentiation potential; ALP, Alzheimer's-like pathology; pct.exp., percentage expression; avg.exp., average expression.

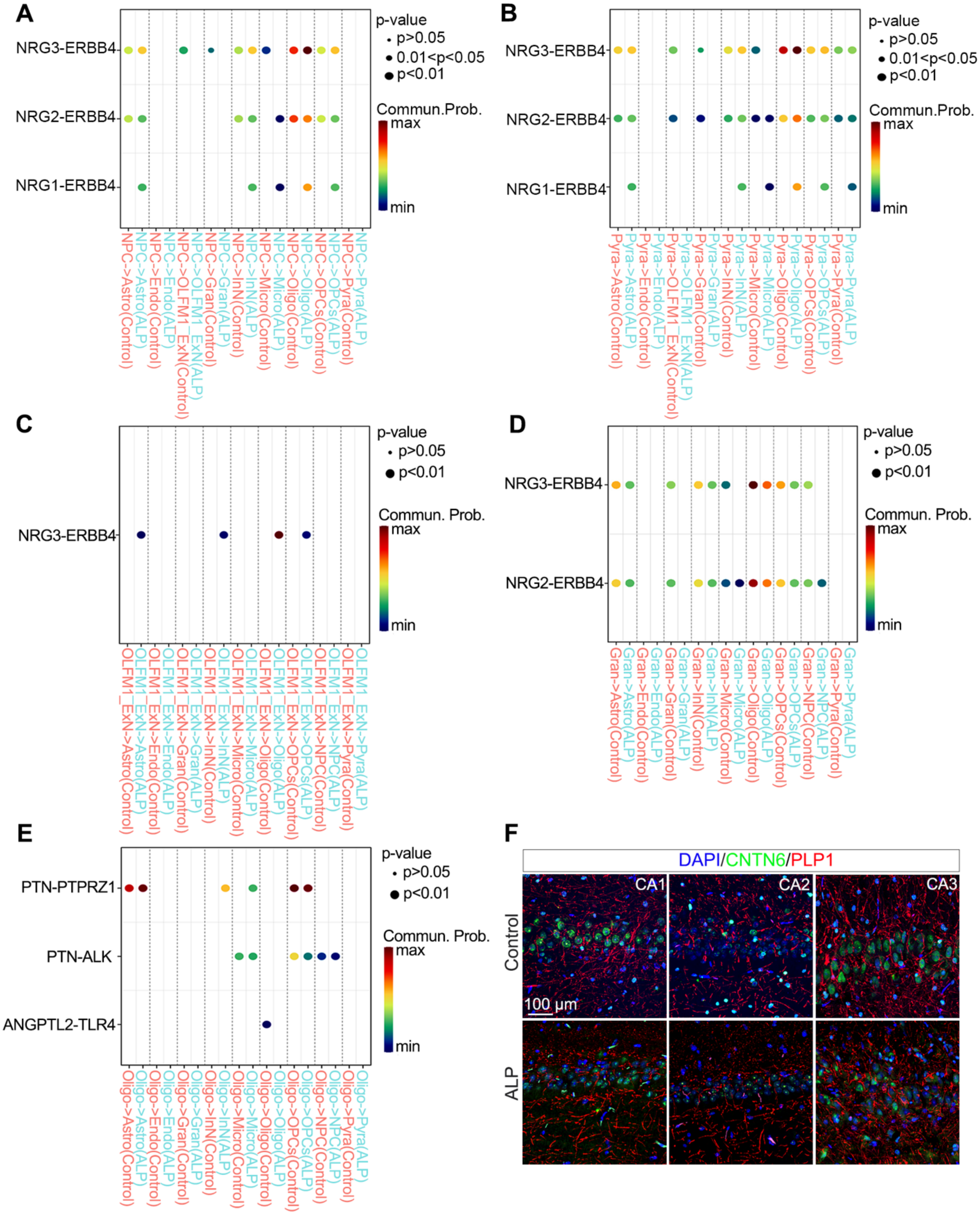

Figure S8 Comparison of the overall relative information flow in each signaling pathway. (A-E) The ligand–receptor interaction strength of cells pairs between ALP and Control groups. Signaling pathways colored red were enriched in the Control group, and those colored green were enriched in the ALP group. (F) Double immunostaining of CNTN6 and PLP1 in the hippocampal

CA1, CA2, CA3 areas of ALP and Control TS. Scale bar = 50 µm. N = 5/group. ALP, Alzheimer's-like pathology; Astro: Astrocytes; Endo: endothelial cells; Micro: microglia; Oligo: oligodendrocytes; OPCs: Oligodendrocyte progenitor cells; Gran: dentate gyrus (DG) neurons; OLFM1_ExN: a subtype of excitatory neurons; Pyra: pyramidal neurons; InN: inhibitory neurons; NPC: neural progenitor cells; Commun.Prob., communications in probability.

Table S1.
ALP tree shrews were selected through pipeline maze, provided with age information.

Table S2.
The number of cells for clusters and cell types.

Table S3.
Marker genes for cell types.

Table S4.
Gene sets used in this study.

Table S5.
snRNA-seq datasets used for cross-species analysis in this study.

Table S6.
The number of cells of snRNA-seq dataset cross species.

Table S7.
Spearman correlation analysis of snRNA-seq dataset cross species.

Table S8.
The antibody information used in this study.

Table S9.
The primer sequences of target genes used in this study.

Materials and methods

Pipeline maze examination

Pipeline maze was innovated in this study to evaluate cognitive function and distinguish between Control and ALP TS. The pipeline maze examination consisted of training and formal testing phases **(Figure 1A)**. In training period, four paths 12A3E4I56, 12A3F4I56, 12A3E4G56, and 12A3E4H56 were sequentially opened, and each TS navigated through them twice. In the formal test, different routes–12A3H4I56, 12A3G4I56, 12A3F4I56, and 12D3E4L56 were opened in turn, with each TS walking through once per route. Testing was conducted at the same time point each day over 4 days, and the time animals spent wandering through the paths was recorded by an individual blinded to the experimental design. ALP and Control groups were determined based on

percentile curves **(Figure 1A)**. The 90% percentile values were calculated as 30 seconds (s) using cumulative frequencies, indicating TS that took longer than 30 s were classified as ALP, while those under 30 s were categorized as Control. Ten TS with longer transit time (>30 s) were assigned into ALP group, while 10 with shorter transit time (<30 s) were arranged into Control group. Finally, 6 TS in each group were selected for subsequent behavioral experiments and imaging examinations. To further verify AD phenotype in TS, the motor, memory, and social abilities of TS were assessed by jumping test, food-induced maze and social function examination.

Jumping test

Jumping test, adapted from our previously established neural behavioral scoring system[1], was employed to test the motor function of the TS hind limbs. Each TS was placed in a box equipped with a height scale, and the times of autonomous standing and the jumping height were recorded and analyzed.

Food-induced maze examination

The food-induced maze examination was used to test the cognitive and memory abilities of TS. The device consists of four transparent pipes connected to each cage and was divided into four zones: A (start), B (transit), C (correct), and D (error) zones. The experiment spanned 3 days, including 2 days of training and 1 day of formal experiment as follows: 1) on 1st day, C zone (containing food) was covered with red cloth, D zone (without food) was covered with green cloth. TS fasted for 8 h were put in the A zone to search for food, with 60 minutes (min) allocated for training; 2) on 2nd day, the roles were reversed: C zone (now without food) was covered with green cloth, D zone (now containing food) was covered with red cloth. TS, again fasted for 8 h, were placed in the A zone for another 60 min of training; (3) on 3rd day (formal test), neither C zone nor D zone contained any food, but were covered with red cloth and green cloth, respectively. Then TS were placed in A zone, and the number of entries into, and time spent in, the C and D zones within 60 min were recorded.

Social function examination

The social function of TS was tested by using a pipeline apparatus, which connected two cages via a tube, allowing free movement of TS between two cages. They were initially placed into their own cages, and over a 24-hour period, the following parameters were observed and recorded: the

number of visits to each other's cage, duration of stay, number of physical contacts, number of active chases, number of passive escapes, sleep time, and wake time.

Positron emission tomography-computerized tomography (PET-CT)

Briefly, 18F-flurodeoxyglucose (18F-FDG)[2] was prepared using GE MINItrace Qilin Medical Cyclotron (GE Healthcare, USA), and TracerLab FDG system (FN model, GE Healthcare, USA), acheving a radiochemical purity of >95%. TS were injected intraperitoneally with 18F-FDG, and key parameters (amount injected, pre-/post-injection time, residual amount) were recorded. PET-CT (Discovery 690/Elite, GE Healthcare, USA) was performed 30 min post-injection. TS was anesthetized with isoflurane and fixed supine for scanning. PET acquisition used 3D mode with a matrix of 128×128, 15-min acquisition time, and 2 bed positions (2.5 min/bed). CT parameters included 120 kV, 260 mA, pitch 0.561, rotation speed 0.5 s, layer thickness 3.75 mm, and field of view 50×50 cm. PET images (47 frames) were reconstructed with MAC mode and attenuation correction. ROIs, including infarcted and contralateral regions, were analyzed using the DICOM network on a post-processing workstation.

Immunohistochemical staining

To investigate the expression of total plaques in the hippocampus (CA1, CA2, CA3 and DG areas) of ALP and Control TS, the immunohistochemical staining was performed. The prepared brain frozen sections were blocked with 3% hydrogen peroxide for 15 min to eliminate endogenous peroxidase, and subsequent incubation with 5% goat serum and 0.3% Triton X-100 was continued for 2 h at room temperature. The sections were then incubated with primary antibody of anti-β-amyloid (6E10, Biolegend, Mouse, 1:1500) at 4°C overnight, followed by incubation with secondary antibodies (MaxVision-HRP, mouse/rabbit) for 15 min. DAB reagent was added to color development. Dehydration with graded ethanol, transparency with TO and sealing with neutral resins were performed continuously. The morphological changes in the brain tissues were observed using a digital pathological section scanner (KF-PRO-005, KFBio, Ningbo, China). Five visual fields (0.5 mm^2 each field) of each section and three sections of each animal were randomly selected to determine the number of Aβ plaques with ImageJ software.

Immunofluorescence staining

The prepared TS brain sections were blocked with 3% hydrogen peroxide for 15 min to eliminate endogenous peroxidase, and subsequent incubation with 5% goat serum and 0.3% Triton X-100 was continued for 2 h at room temperature. The sections were then incubated with primary antibodies against APP (Servicebio, Rabbit, 1:200), and p-Tau (Abcam, Rabbit, 1:200) at 4°C overnight, followed by incubation with secondary antibodies. The information of antibodies was provided in **Table S8** in detail. The sections sealed with a DAPI-contained quencher were observed under the two-photon confocal microscope (Leica TCS SP8 DIVE) at x400. The morphological changes in the brain tissues were observed using a digital pathological section scanner (NIS-Elements AX). Five visual fields (2048×2048 pixels) of each section and three sections of each animal were randomly selected to determine the integrated intensity of APP and p-Tau (S404) with ImageJ software.

Multiplex immunofluorescence staining

Hippocampus sections of Control and ALP TS were prepared for multiplex immunofluorescent staining. The tissues were dissected and fixed with 4% paraformaldehyde for up to 24 h and cryoprotected in 30% sucrose at 4 °C for 72 h. The tissue samples were frozen with optimal cutting temperature compound (Tissue-Tek) at −80 °C for 30 min and sectioned at a thickness of 15 μm using a microtome (Leica CM1950). Sections were then stored at −80 °C. After antigen repair with sodium citrate, the sections were incubated with H_2O_2 for 10 min and blocked with 5% goat serum and 0.3% Triton X-100. Subsequently, the sections were supplemented with primary antibodies for 18 h at 4°C. This was followed by rinsing in PBST five times (5 min each). Secondary antibodies were added, followed by incubation at room temperature for 15 min. This was followed by washing with PBST and incubation for 10 min with TSAPlus fluorescent enhancement dye. For multiplex staining, these sections were subjected to antigen repair again with sodium citrate and were incubated with H_2O_2 for 10 min, followed by blocking with 5% goat serum and 0.3% Triton X-100. Incubation with primary and secondary antibodies was performed as described previously. Finally, after counterstaining with DAPI for 10 min, sections were sealed with an anti-fluorescent quenching agent. Using a two-photon confocal microscope (NIS-Elements AX), five visual fields (2048×2048 pixels) of each section and three sections of each group were randomly selected to quantify the positive cells, and the number of positive cells was calculated using the ImageJ software. Detailed antibody information is provided in **Table S8**.

Nissl staining

Nissl staining was performed to detect total and dark neurons in the cortex and hippocampus (CA1, CA2, CA3, DG) of both ALP and control TS. Sections were stained for 10 minutes at room temperature, differentiated in alcohol, and cleared with turpentine oil. Images were captured using a digital scanner (Pannoramic MIDI, x100), with five visual fields per section analyzed for neuron count and dark neuron ratios using ImageJ.

GO and KEGG pathway enrichment Analysis

The ‘‘enrichGO()” or ‘enrichKEGG()” function of cluster Profiler R package[3] was used for GO or KEGG enrichment analysis, and the Benjamini-and-Hochberg (BH) method was employed for multiple test correction. A GO term or KEGG pathway with an adjusted P-value lower than 0.05 was considered as significantly enriched.

Trajectory analysis

Monocle3 (v.1.2.9) were used to infer the cells state transition. A CellDataSet object was created from Seurat analysis. DDRTree method and orderCells function were used for dimensional reduction and ordering cells, respectively. NPC populations were regarded as the root state of the trajectory inference.

Cell–cell interaction analysis

Cell–cell interactions among different cell types were computed by CellChat (v.1.1.3). In order to perform CellChat analysis in TS, the genes of TS were converted to human genes based on homologous gene mapping. Then, comparison analysis between ALP and Control groups was performed by using CellChat. The differential number of interaction strength in the cell-cell communication network between ALP and Control groups was visualized by using circle plot. Differential interaction strength was also shown by using heatmaps. By comparing the information flow of each signaling pathway, we identified signaling pathways. We also compared the communication probabilities mediated by ligand-receptor pairs from some cell populations to other cell populations.

References

1. Wang YY, Niu RZ, Wang JD, Jin Y, Wang TH, Liu F. Establishment of brain ischemia model in tree shrew. *Brain Res*. Sep 1 2019;1718:194-200. doi:10.1016/j.brainres.2019.05.011
2. Hansen S, Keune J, Küfner K, et al. The congruency of neuropsychological and F18-FDG brain PET/CT diagnostics of Alzheimer's Disease (AD) in routine clinical practice: insights from a mixed neurological patient cohort. *BMC Neurol*. Mar 9 2022;22(1):83. doi:10.1186/s12883-022-02614-4
3. Lee KE, Seo J, Shin J, et al. Positive feedback loop between Sox2 and Sox6 inhibits neuronal differentiation in the developing central nervous system. *Proc Natl Acad Sci U S A*. Feb 18 2014;111(7):2794-9. doi:10.1073/pnas.1308758111
